# A Comparative Pilot Study of Bacterial and Fungal Dysbiosis in Neurodevelopmental Disorders and Gastrointestinal Disorders: Commonalities, Specificities and Correlations with Lifestyle

**DOI:** 10.3390/microorganisms9040741

**Published:** 2021-04-02

**Authors:** Ibrahim Laswi, Ameena Shafiq, Dana Al-Ali, Zain Burney, Krishnadev Pillai, Mohammad Salameh, Nada Mhaimeed, Dalia Zakaria, Ali Chaari, Noha A. Yousri, Ghizlane Bendriss

**Affiliations:** 1 Premedical Education Department, Weill Cornell Medicine Qatar, Doha 24144, Qatar; ikl2001@qatar-med.cornell.edu (I.L.); abs2024@qatar-med.cornell.edu (A.S.); dka2003@qatar-med.cornell.edu (D.A.-A.); zab2004@qatar-med.cornell.edu (Z.B.); kvp2002@qatar-med.cornell.edu (K.P.); mas2207@qatar-med.cornell.edu (M.S.); nam2053@qatar-med.cornell.edu (N.M.); dez2003@qatar-med.cornell.edu (D.Z.); alc2033@qatar-med.cornell.edu (A.C.); 2 Research Department, Weill Cornell Medicine Qatar, Doha 24144, Qatar; nay2005@qatar-med.cornell.edu; 3 Computers and System Engineering, Alexandria University, Alexandria 21526, Egypt

**Keywords:** dysbiosis, microbiome, bacteriome, mycobiome, neurodevelopmental disorders, gastrointestinal disorders

## Abstract

Gastrointestinal disorders (GIDs) are a common comorbidity in patients with neurodevelopmental disorders (NDDs), while anxiety-like behaviors are common among patients with gastrointestinal diseases. It is still unclear as to which microbes differentiate these two groups. This pilot study aims at proposing an answer by exploring both the bacteriome and the mycobiome in a cohort of 55 volunteers with NDD, GID or controls, while accounting for additional variables that are not commonly included such as probiotic intake and diet. Recruited participants answered a questionnaire and provided a stool sample using the Fisherbrand collection kit. Bacterial and fungal DNA was extracted using the Qiagen Stool minikit. Sequencing (16sRNA and ITS) and phylogenetic analyses were performed using the PE300 Illumina Miseq v3 sequencing. Statistical analysis was performed using the R package. Results showed a significant decrease in bacterial alpha diversity in both NDD and GID, but an increased fungal alpha diversity in NDD. Data pointed at a significant bacterial dysbiosis between the three groups, but the mycobiome dysbiosis is more pronounced in NDD than in GID. Fungi seem to be more affected by probiotics, diet and antibiotic exposure and are proposed to be the main key player in differentiation between NDD and GID dybiosis.

## 1. Introduction

Dysbiosis, or the alteration in the diversity and abundance of the gut microbial communities, has been consistently linked to modern diseases such as metabolic diseases [1,2], neurodegenerative diseases [3], cancers [2], autoimmune diseases [4] and even neuropsychiatric and neurodevelopmental disorders (NDD) such as Autism Spectrum Disorders (ASD) and Attention Deficit Hyperactivity Disorders (ADHD). Furthermore, it has been shown that a significant proportion of children with NDD often also suffer from gastrointestinal disorders (GID) such as celiac disease, irritable bowel syndrome or inflammatory bowel diseases (IBD) [5]. Interestingly, studies noticed that individuals with a GID also often develop anxiety-like behaviors [6,7,8]. This has been described as the gut–brain axis or GBA. A growing number of studies have greatly emphasized this bidirectional communication between the gut and the brain as well as the role of microbes and the metabolome in the pathogenesis of anxiety-like behaviors [9,10,11,12]. Microbiome alterations have been associated not only with ASD, ADHD, schizophrenia and anxiety, but also with Crohn’s diseases, ulcerative colitis and celiac disease [13,14,15]. These observations have led to the hypothesis that a general dysbiosis exists in both types of disorders, but while some features might be shared, others might certainly differentiate them.

Most studies that have investigated the dysbiosis in Autism and IBD have done it separately and have never reached a consensus on which microbial species are significantly affected and the relationship between the two types of disorders. In addition, replicating the results remained difficult, since the source of the sample (colon, stomach, mouth), lifestyle (diet, probiotic intake, antibiotic history) and method of collection are factors that affect both relative and absolute abundances of gut organisms and ultimately our understanding of gut composition. Moreover, despite the microbiome being a rich and complex ecosystem that includes not only bacteria but also fungi, protists, viruses and archaea, most studies to date have been devoted to the bacteriome while completely neglecting the mycobiome. Fungi are an integral part of the human flora and can be found in the gut as well as the skin, vagina, mouth and lungs [16,17,18]. Interestingly, recent studies have pointed to the importance of the mycobiome in ASD and a possible interaction between the bacteriome and mycobiome [16,19,20]

A variety of external factors have been described as modulators of the gut microbial composition, most of them pertain to lifestyle and include diet, physical activity, sleep, and stress [3,21,22,23,24]. Studies have identified a critical window for the gut microbiota maturation during infancy, and during which the drastic alterations of the gut microbiota could lead to further dysbiosis [25,26,27,28]. Processed carbohydrates such as white flour, white bread, white rice, pastries, pasta, sweets, breakfast cereals, added sugars, soda and snacks have been shown to greatly affect the microbiome composition [29]. Interestingly, this group of food seems to be craved by individuals with mood disorders. 

In addition to lifestyle, probiotics and antibiotics have direct and indirect impacts on the growth of gut microbes. Probiotics intake has recently become a trend among individuals with health concerns due to marketing and increasing awareness of the importance of gut microbes and this can also directly affect the observed microbiome composition [30]. Indeed, studies have pointed to the important role of probiotics in re-establishing an eubiosis after a dysbiosis in diseases of the GBA [31,32,33]. A recent study by Lukacik et al. [34] has mentioned the possible role of early life exposure to antibiotics in the risk of developing autism. Slob et al. [35] showed in a twin study on 27,781 twins that early exposure to antibiotics significantly increased the risk of developing ADHD and ASD. This intriguing association between NDD and early exposure to antibiotics has been shown by a growing number of studies [34,36,37,38]. These three variables are not commonly taken into consideration when studying the gut microbiome.

In Qatar, the prevalence of ASD has been estimated at 1.81%, which is higher than world average, which is 0.6 to 1% [39]. However, no study has investigated the microbial signatures of individuals with NDD and/or GID in Qatar. Our previous questionnaire-based study assessed the awareness of both the general public and healthcare professionals in Qatar on the role of gut microbiome in NDD and GID and attempted to understand some of the dietary habits of 112 participants and their early exposure to antibiotics. The study showed an association between early exposure to antibiotics (before age 3) and developing an NDD [40]. Fifty-five of those participants volunteered to also donate a stool sample for the present analysis.

As the interest in the mechanisms underlying pathologies relating to the GBA is growing worldwide, comparing the gut composition of NDD and GID is essential to further understand the relationship between these two sets of disorders. In addition, exploring both the bacteriome and the mycobiome is becoming essential, as is the role of the external modulators.

The goal of this pilot study is therefore to analyze and compare both the gut bacteriome and mycobiome among individuals with NDD, GID, and non-NDD/non-GID controls (CTRL) of a small cohort in Qatar. This study attempts to identify microbial signatures of both bacteria and fungi in NDD and GID compared to controls, while taking into consideration antibiotic history at early age, diet and probiotic intake. Launched in 2018, this is the first study that compares both the bacteriome and mycobiome of individuals with NDD or GID with healthy individuals within the same cohort of a multiethnic population, while taking into consideration these external modulators of the gut microbiota.

## 2. Materials and Methods

### 2.1. Recruitment

Recruitment was performed using an Institutional Review Board (IRB)-approved flyer calling for volunteers, which was shared on social media and by email. Recruitment also happened during a seminar presentation of the project and during the Autism Family Day at a center for special needs in Qatar. Fifty-five participants were recruited according to the following inclusion criteria: 3 to 90 years old (however, only 3 to 57 volunteered) living in Qatar and experiencing one of the following conditions: ASD, ADHD (both included in NDD); Crohn’s disease OR ulcerative colitis OR celiac disease (all included in GID); or none of the previously mentioned conditions (CTRL). Exclusion criteria were: the participant or parent giving consent is able to read neither English nor Arabic. Participants were asked whether they have been taking antibiotics in the past month prior to stool collection, and none of them had. The recruited number of participants (*N*) per group were as follows: NDD: *N* = 15; GID: *N* = 13 and C: *N* = 27.

Consent forms were written in both English and Arabic. Non-cognitively impaired adults had a consent form to fill and sign. For children and cognitively impaired adults and children, the recruitment required consent from one or both parents and the child’s assent. Two different assent forms were designed and approved by the IRB: one for non-cognitively impaired children and one for the cognitively impaired children/adults. These forms aimed to ensure the participant understands that they will be providing a stool sample and how it will be used.

### 2.2. Questionnaire

A survey collected personal data for statistical correlation analyses: age, gender, height, weight, disease group (NDD, GID, CTRL), diet type, medical history (presence of other diseases, extensive use of antibiotic during first three years of age), ethnicity. 

### 2.3. Sample Collection

Participants were offered a “commode specimen collection system” (Fisherbrand) to collect their own or their child’s stool sample at home. The Fisherbrand collection kit was chosen for its intuitive, safe and easy collection of a stool sample for both adults and kids without requiring the participant to handle the stool. The kit is placed on the toilet bowl and closed with a cap after defecation. Alphanumeric labels were added to each sample and to a corresponding survey upon reception. 

### 2.4. DNA Extraction

QIAamp Fast DNA stool mini kit (Qiagen, Hilden, Germany) was used to extract DNA from fresh stool samples. Stool samples were vortexed and sonicated for 5 min prior to following the manufacturer’s protocol. Samples were lysed in InhibitEX Buffer, then incubated at 90 °C. After centrifugation at high speed, the bacterial genomic DNA was purified on the QIAamp Mini spin columns as follows: protein digestion by proteinase K occurred at 70 °C incubation then loaded on the QIAamp spin columns for silica membrane DNA binding, washing steps, and elution of DNA. Short centrifugation at high speed allowed adsorption of the DNA on the membrane. Two washing steps preceded the elution, which used a low-salt buffer suitable for direct use of the DNA for PCR reactions. Storage of purified DNA was done at −20 °C. DNA quality and quantity were assessed using a nanodrop spectrophotometer and measurements of its absorbance at 260 nm and 280 nm. The minimum concentration required was set to 100 ng with at least 20 µL of volume.

### 2.5. 16sRNA Sequencing and Phylogenetic Analysis

All amplified DNA samples were sent to CD genomics (Shirley, NY, USA), which performed 16sRNA sequencing and phylogenetic analyses using the PE300 Illumina Miseq v3 sequencing with a 600-cyle format. Standard analysis included operational taxonomic unit (OTU) clustering, alpha-diversity analysis, OTU analysis/species annotation (phylogenetic tree, Venn graph, Heatmap, and taxonomic tree), beta-diversity analysis (PCA analysis, PCoA analysis, unweighted UniFrac distance heatmap, UPGMA and NMDS analysis). CD genomics provided a full report with raw data excel files.

### 2.6. ITS Sequencing and Phylogenetic Analysis

Sequencing and analysis of the internal transcribed spacers (ITS) was used by CD genomics to analyze the fungal composition of samples. After testing the DNA quality required for ITS sequencing, only 35 (out of 55) samples were retained for fungal DNA analysis: NDD: *N* = 8; GID: *N* = 11; CTRL: *N* = 16. Standard phylogenetic analysis included the same analyses as for 16sRNA listed above and reports, and raw data were sent to us for further analyses. 

### 2.7. Statistical Analyses

Data including age, gender, body mass index (BMI), history of probiotic use, history of antibiotic use in the first three years of life, and nature of diet were collected as described in Section 2.2. The , or diet, or both, in addition to basic covariates. A Student’s *t*-test was used to compare abundances among individuals based on their antibiotic use in the first three years of life. *p*-values < 0.05 were considered significant. All analyses were completed in R statistical package version 4.0.2.

## 3. Results

### 3.1. Characterisistics of the Studied Cohort

Table 1 depicts the three groups’ characteristics based on the data collected from the surveys. Besides the cosmopolitan recruited population, most participants with NDD were from the Middle East, while most participants with GID were caucasian. Although we did not have run a test on this, these interesting numbers are in concordance with previous studies pointing at racial differences in GID occurrence. 

### 3.2. Bacterial Microbiome in NDD, GID and CTRL

#### 3.2.1. An Altered Bacterial Diversity in NDD and GID Groups

Observations at the phylum level note various phyla ratios that are significantly changed in NDD compared to CTRL and compared to GID (Table 2). In particular, the ratio of Firmicutes to Bacteroidetes is significantly decreased in NDD compared to CTRL (*p* = 0.045), but not significantly different between NDD and GID (*p* = 0.87, data not shown), which indicates that NDD and GID groups are closer in regard to this specific ratio. Another important ratio is the Firmicutes to Actinobacteria, which is significantly decreased in NDD compared to both CTRL and GID. Other significantly changed ratios are listed in the supplementary file.

In the GID group, the ratios involving TM7—a recently described subgroup of uncultivable bacteria also called Sacharibacteria—with a decreased ratio of TM7/Actinobacteria (*p* = 0.041) compared to CTRL, and a decreased TM7/Firmicutes ratio (*p* = 0.044). These are in concordance with previous studies associating TM7 with IBD [41].

**Figure 1:**
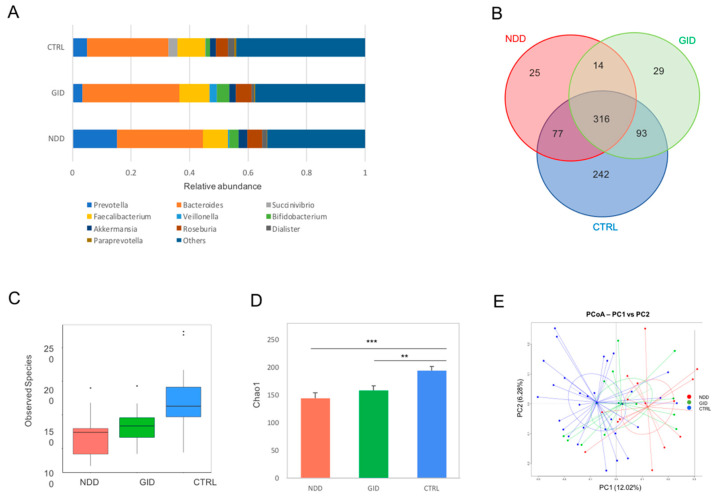
Overview of bacterial diversity in NDD, GID and control (CTRL). Relative abundances of the 11 most significant bacterial genera (**A**); Venn diagram summarizing number of operational taxonomic units (OTUs) shared between different groups (**B**); alpha diversity indices including the number of observed species (**C**) and the Chao1 index (**D**); beta-diversity indicated by PCoA plot of unweighted UniFrac distance by groups (**E**). Significant difference of the NDD group compared to CTRL and GID group compared to CTRL was noted by * *p* < 0.05; ** *p* < 0.05; and *** *p* < 0.005.

While ratios analyses can indicate a level of dysbiosis, these are not informative enough, and in fact, some ratios can still be similar while the abundance of both taxa involved is increased or decreased. Therefore, alpha diversity refers to the diversity within each sample; the richness is indicated by the number of observed species and the Chao1 index (estimation based on abundance). Both are represented in Figure 1. The representation in Figure 1A at the genus level points at a progressive shift from CTRL to GID to NDD, with shifts being more pronounced in NDD than GID. The Venn diagram confirms that the number of operational taxonomic units (OTUs) shared by the GID group and CTRL group is higher than NDD with CTRL, while both NDD and GID groups appear to be characterized by some unique OTUs (Figure 1B). The number of observed species is significantly decreased in both NDD and GID groups compared to the CTRL group, and the Chao1 index indicates that this decrease is more significant in NDD than in GID (Figure 1C,D). 

The beta diversity indicated by the performed principal coordinated analysis (PCoA) based on the weighted UniFrac analysis of the OTU level revealed that the overall composition of the NDD bacteriome is different from that of the CTRL group, while the diversity of the GID group stands somewhere in between CTRL and NDD (Figure 1E).

#### 3.2.2. Significant Taxa Abundance Variability in NDD Compared to CTRL

Table 3 and Table 4 depict the respective measurements of absolute abundance and relative abundance of taxa across phylogenetic levels in the NDD group compared to CTRL. Some differences in taxa are only significant when using relative abundance. For example, the significant decrease in Firmicutes in NDD is only observed while using relative abundance; the increase in the order of Clostridiales in NDD is also significant when only using relative abundance. Three more families, one more genus and three more species appear to be significant when using relative abundance.

In addition, statistical models using basic covariates (BMI, gender, age) or including the intake of probiotics or the type of diet also influence the results and some taxa showing significant differences with basic covariates become non-significant once we considered the use of probiotics or the diet or both. For example, the species *Bifidobacterieum bifidum* and *Veillonella dispar* seem to have a significantly increased abundance in NDD, but not if probiotics intake and diet are considered. On the contrary, the increase in the family of *Corynebacteriaceae* and the genus of *Odoribacter* is only significant when considering relative abundance and the intake of probiotics and diet.

Therefore, the *p*-values of absolute abundance with the most stringent model (*p*-value 3), including basic covariates as well as probiotics and diet, will be considered in our following conclusion. However, it is still important to mention the abundances that do not take those into account, as well as the relative abundance for comparison purposes with other studies.

To conclude this section about bacteriome in NDD, the NDD group shows no significant changes at the phylum level and class level when considering absolute abundance and when including probiotic intake and diet in the covariates. As a result, these are the following taxa that are significantly changed in NDD compared to CTRL: orders of Enterobacteriales and RF32, the family of *Enterobacteriaceae*, genera of *SMB53*, *Escherichia*, *Clostridium*, *Butyricicoccus*, *Enterococcus*, *Lactococcus*, *Veillonella*, *Coprococcus*, which all increase except for *Coprococcus,* which decreases in NDD. Interestingly, the relative mean abundance of Prevotella (Figure 1A) suggest a possible increase in NDD. However, this is not significant with either of the models, even when using relative abundance (data not shown). 

#### 3.2.3. Comparing NDD Group to GID Group: Commonalities and Specificities in the Bacteriome

Table 5 shows that the GID group holds only a limited number of significant changes in bacterial abundances compared to CTRL. Interestingly, apart from SMB3, *Echerichia coli* and Anaerotruncus, which are increased in NDD compared to GID, and Odoribacter, which is decreased in NDD, no other significant changes are noticed between NDD and GID (Table 6). Table 4, Table 5 and Table 6 depict only a limited number of significant changes in GID compared to the CTRL group, as well as a limited number of significant changes in GID compared to the NDD group. 

Added to the alpha and beta diversity data observed in Figure 1, this suggests that the bacterial diversity of the GID group lies somewhere between the CTRL and the NDD groups and that the bacterial dysbiosis observed in GID is less pronounced than in the NDD group.

### 3.3. The Fungal Microbiome or Mycobiome: A Neglected but Important Player

#### 3.3.1. An Increased Fungal Diversity in the NDD Group

**Figure 2:**
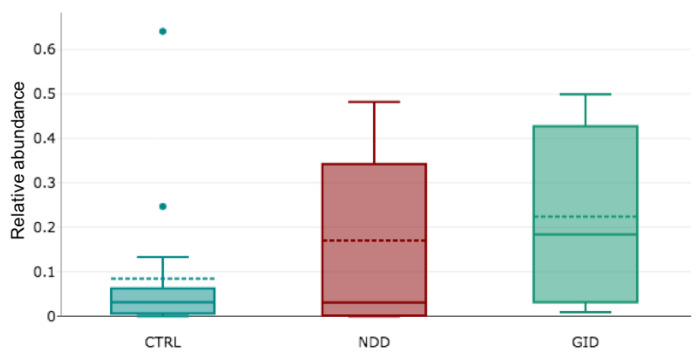
Box plot of relative abundance of Saccharomyces genus in CTRL, NDD and GID groups.

The fungal microbiome or mycobiome includes two main phyla, which are the Ascomycota and Basidiomycota. Other phyla are Chytridiomycota, Mucoromycota, Mortierellomycota. *Saccharomyces* genus is so preponderant that its abundance makes visualization of other genera difficult. For this reason, we have separated them: Figure 2 depicts the relative abundance of *Saccharomyces* in CTRL, NDD and GID groups and shows a significant increase of its abundance in the NDD group (*p*-value = 0.0044; Appendix A, Table A1).

**Figure 3:**
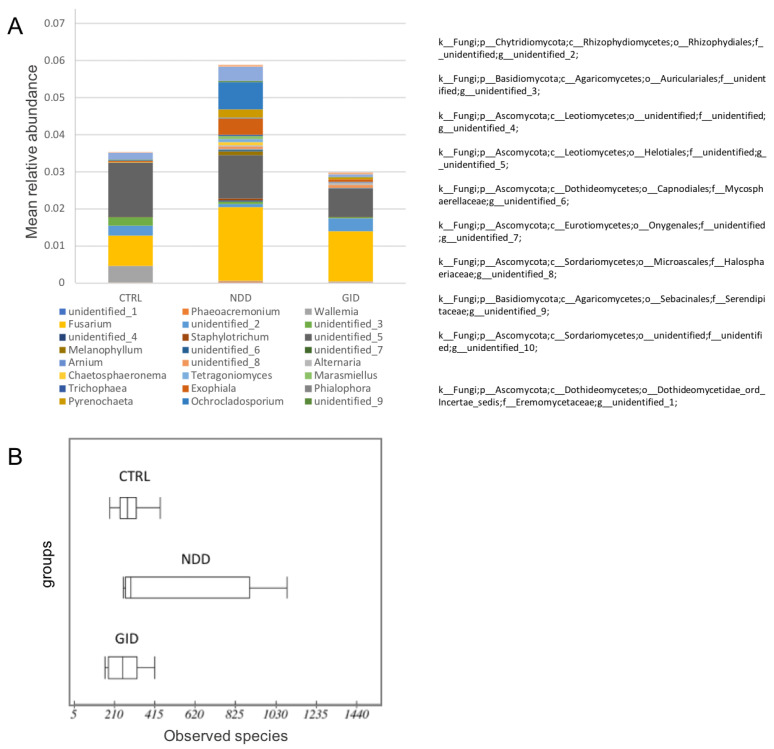
Fungal alpha diversity in CTRL, NDD and GID. Mean relative abundances of 14 most significant fungal genera and the higher taxonomic classification of the unidentified genera (**A**); alpha diversity by the number of observed species (**B**).

Figure 3 shows the mean relative abundance of the 14 most significant fungal genera in NDD, CTRL and GID. Interestingly, 10 of those genera are unidentified: one of them being from the Chytridiomycota phylum, two others from the Basidiomycota phylum and the rest from the Ascomycota phylum. We note that the fungal diversity is actually increased in NDD compared to both CTRL and GID, which might indicate the overgrowth of certain fungal species (Figure 3A,B).

#### 3.3.2. An Increased Fungal Abundance in the NDD Group

Significant changes in abundance of the mycobiome community in NDD were observed at all taxa levels (Appendix A Table A1). Among the 28 genera that significantly changed, *Saccharomycces* is noted as one of the most significant genera for which the abundance increases in NDD (Appendix A Table A1). Interestingly, the sequencing noted 15 unidentified species that changed significantly in NDD (nine significantly increased; six significantly decreased). Most of the unidentified species are from the Ascomycota phylum (11 out of 15), two are from the Basidiomycota phylum, one is from the Mortierellomycota phylum and one is from the Chytridiomycota phylum.

In the previous section, *p*-values of bacterial abundances did not seem to greatly differ, as we included additonal variables into the model (probiotics intake and diet). However, fungi abundances seem to be greatly affected by the intake of probiotics and the type of diet, as including those variables resulted in the appearance/disappearance of a higher number of significantly different abundant taxa (Appendix A Table A1). For this reason, the following tables depict absolute abundance using the most stringent model. 

Table 7 lists the 26 species with significantly increased abundance in NDD compared to CTRL and the 10 species with significantly decreased absolute abundance. 

#### 3.3.3. Mycobiome Is Key to Differentiate between NDD and GID Dysbiosis

As described in the previous section, the bacteriome of NDD and GID groups only showed significant differences in three genera and one species. However, the exploration of the mycobiome shows that, unlike for the bacteriome, NDD and GID show distinct fungal communities. Indeed, the regression analysis across all fungal taxa shows a significant change in abundance in NDD compared to GID in 1 Phylum, 4 classes, 4 orders, 5 families, 8 genera, and 10 species of fungi. Table 8 depicts all significant changes at each taxa level.

On the contrary, only 10 species are significantly increased in the GID compared to the CTRL group (Table 9), supporting the hypothesis of a GID mycobiome diversity that is closer to the CTRL group than the NDD group. 

Several of our participants (three CTRL, two NDD and three GID) reported suffering from recurrent mucosal or dermal Candida infections (data not shown). Although the most reported type of candidiasis is by *Candida Albicans*, this species did not appear in this analysis as being significantly changed, neither in NDD nor in GID. However, the species *Candida parapsilosis* was found to be significantly increased (*p* = 0.0159) in GID compared to CTRL. 

### 3.4. Exposure to Antibiotics at Early Age Is Associated with a Significant Fungal Dysbiosis 

#### 3.4.1. Effect of Recurrent Exposure to Antibiotics before 3 Years: An Independent *t*-Test

As our participants who provided stool samples also have answered a question regarding their exposure to recurrent courses of antibiotics before age 3, we were interested in exploring dysbiosis in those two groups: exposed (+ABX) or not exposed (−ABX). The independent T-test was run for both bacteria and fungi, using absolute and relative abundances, and at all taxa levels. Only results using absolute abundance are shown in Table 10. Interestingly, results indicate that exposure to ABX is associated with greater fungal dysbiosis than bacterial dysbiosis. Indeed, for bacteria, only one phylum, one class, one family, four genera and two species are significantly different. However, for fungi, 1 phylum, 2 classes, 4 orders, 6 families, 10 genera and 12 species show a significantly different abundance between the two groups.

For this reason, to explore further, a regression analysis for the new variable ABX was performed, while accounting for the basic covariates, probiotic intake, diet and the group NDD or GID. Table 10 below shows the *p*-value for the use of antibiotics before 3Y in NDD group for both bacteria and fungi.

Table 10 shows the *p*-value for the use of antibiotics before 3Y in the GID group for both bacteria and fungi.

Whether in NDD or GID, antibiotics seem to be associated with more fungal dysbiosis than bacterial dysbiosis, as more taxa are affected in the fungi kingdom.

#### 3.4.2. Regression Analysis for the Effect of ABX

The analysis noted that while taking into account the NDD group in addition to the basic covariates and probiotics and diet, the abundance of two bacterial families, eight bacterial genera and two bacterial species are significantly changed when exposed to ABX at an early age, and that two fungal phyla, 4 fungal orders, 4 fungal families, 11 fungal genera and 12 fungal species (two of them non-identified) are significantly affected as well (Table 11). 

Therefore, although the regression model results in some different taxa involved compared to the *t*-test, it still confirms that early ABX exposure does affect the fungi kingdom in NDD and maybe even more than bacteria. 

The same pattern is noticed for GID (Table 12). Indeed, only two bacterial genera—*Atopobium* and *Allobaculum*—and one bacterial species—*eggerthii*—were affected by introducing the ABX exposure variable. However, 2 fungal phyla, 3 classes, 3 orders, 6 families, 11 genera and 13 species of fungi are significantly associated with early ABX exposure. 

## 4. Discussion

### 4.1. From GID Group to NDD Group: What Do They Have in Common, and in What Ways Are They Different?

Several studies have shown that gut dysbiosis in both ASD and IBD is characterized by a decrease in biodiversity [42,43,44]. The present study also noted that the bacterial alpha diversity is significantly decreased in both NDD and GID groups compared to the CTRL group, but more significant for the NDD than the GID group. In the GID group, *Streptococcus luteciae* was the only significant species increasing compared to CTRL. A study by Liang et al. [45] also showed in a model of colitis-associated colorectal cancer that this species was increased during the process of carcinogenesis. However, *E. coli* was shown to be increased in our NDD cohort, while some studies showed the opposite [46]. As compared to the NDD group, the bacterial dysbiosis observed in GID only showed significant differences in three genera and one species, and all other taxa were not significantly different. Interestingly, this GID group also exhibited only a limited number of significant changes compared to the CTRL group. This suggests that the GID bacteriome is somewhere between the CTRL and the NDD and that only a limited number of taxa seem to differentiate between the groups. 

This raised the following question: is there another gut component that presents more significant changes between the groups? This study provides preliminary data that support the following hypothesis: GID and NDD share a similar bacteriome dysbiosis but have a different mycobiome. Indeed, while the alpha diversity decreases for bacteria in both NDD and GID groups, it rather significantly increases for fungi in the NDD group (Figure 1C and Figure 3B). Moreover, the regression analysis across all fungal taxa showed a significant change in abundance in NDD compared to GID group across all taxa levels, with 1 Phylum, 4 classes, 4 orders, 5 families, 8 genera and 10 species of fungi. Interestingly, the comparison of Table 8 and Table 9 suggests that the GID group is characterized by an increased abundance in 10 fungal species compared to CTRL and eight different other species compared to NDD group, suggesting that the fungal dysbiosis in GID might be key to understanding both the development of GID and NDD.

One of the identified fungi that is significantly increased in GID is *Saccharomyces cereviciae (S. Cereviciae)*. A recent study by Torres et al. [47] has identified antibodies against *S. Cereviciae* as one of the 51 biomarkers that can successfully predict the diagnosis of Crohn’s disease within 5 years with high accuracy. We also showed a significant increase in *S. Cereviciae* in the NDD group, as shown by other studies [33]. This suggests that the autoimmunity against this specific species might be a good predictor of those individuals in the NDD group who will also develop severe GID [48]. 

The presence of 15 unidentified fungal species whose abundance significantly changes in NDD not only emphasizes the possible crucial role of fungi in microbiome dysbiosis and pathogenesis in NDD but also testifies of the need for more studies on the mycobiome. Indeed, studies have shown that only a limited number of internal transcribed spacer DNA sequences are available on GenBank [49]. Recent findings suggest that there is competition between bacteria and fungi and that prolonged antibiotic use is associated with fungal infections [50,51]. Therefore, fungi seem to be key in understanding the spectrum going from simple gastrointestinal disorders to more complex neurodevelopmental disorders that combine with gastrointestinal disorders. Fungi might also be key in understanding the spectrum of symptomatology in ASD. While this study is only a pilot study with a relatively small sample size, it points to important directions to explore further to understanding pathogenesis of NDD and GID.

### 4.2. Need for Standard Measurements and Limitations of the Study

Data suggest that there is a need for establishing measurement standards in order to reveal the microbial communities that play a key role in pathogenesis of the diseases. 

In addition to methods of extractions, sample origin and bacterial culture, data suggest that the use of relative vs. absolute abundance as well as including some factors related to lifestyle such as probiotic intake or diet can lead to large discrepancies between data obtained from various studies [52,53]. While the gold standard approach to accurately determine taxonomic shifts is to use absolute abundance [54], most studies replace this laborious requirement by the use of the relative abundance. In this study, both relative and absolute abundances were computed to allow for comparisons with other studies, and four different regression models were used to include some additional variables. Studies have shown that the delivery mode, exclusive breastfeeding, stress, smoking, sleeping and exercise are also important factors that have been associated with changes in microbiome composition and could also be used as additional variables [55]. Our study only looked at probiotic intake and diet. Some examples of the discrepancies found are as follows: the increase of the order of Clostridiales in NDD is only significant when using relative abundance. The species *bifidum* and *dispar* seem to have a significantly increased abundance in NDD, but not if probiotics intake and diet are considered. Studies have indeed shown that *bifidum* is actually decreased in children with ASD [56,57]. Similarly, the increase in the family of *Corynebacteriaceae* and the genus of *Odoribacter* is only significant when considering relative abundance and the intake of probiotics and diet. This emphasizes that not considering lifestyle in microbiome studies could lead to misinterpretations and be a barrier to finding microbial signatures.

While investigators have ensured that none of the participants were exposed to antibiotics a month before the stool collection, another limitation of this study to consider is that they did not ask participants whether they had been taking an anti-fungal in the past months prior to the stool collection. Although the intake of anti-fungals among children is not as common as intake of antibiotics, the observed decreased abundance of some fungal species in NDD must take that into account.

In addition, it is important to note that these observations among GID and NDD groups were performed on different age groups (Table 1). Indeed, most NDD participants were between 3 to 18 years old, and most GID participants were between 19 to 60 years old. Nevertheless, studies observed in healthy individuals that the gut microbiota is stabilized in an adult-like configuration after the first 3 years of life [28,58,59,60]. A study by Nagpal et al. [61] showed that the most significant change in microbiome composition happens during the window of 0 to 3 years after birth. Furthermore, the decline in microbiome diversity that occurs with age has been described by studies to be a consequence of several lifestyle changes, including nutrition [62]. Since age and lifestyle are confounding factors, it is very difficult to separate the effect of aging from the dietary change effect on the gut microbiome. Due to these reasons, this pilot study did not consider the age variable in its statistics but considered an aspect of the diet.

### 4.3. Effect of Probiotics and Diet 

Fermented foods, such as cultured milk products and yogurt, provide the body with ingestible microorganisms that may modulate the intestinal homeostasis and treat or prevent IBD [63]. Consumption of probiotics was reported to increase the abundance of *Bifidobacteria* and/or *Lactobacilli* and to reduce the counts of *E. coli* and *Helicobacter pylori*. Furthermore, they regulate inflammation by reducing the proinflammatory cytokine IL-6, and increasing the anti-inflammatory cytokine IL-10. Probiotics were also found to increase the total serum IgA, which potentiates the humoral immune response. In addition, they inhibit the adhesion of pathogens to the intestinal mucosa and promote the production of SCFA [64].

In this study, it was observed that higher proportions of the NDD group consumed a diet with medium to high processed carbohydrates as compared to the GID and CTRL groups. It has been well established that carbohydrates can modify the gut microbiome [65,66]. Artificial sweeteners, often present in processed food, have also been shown to alter the gut microbiota [67,68]. A growing number of studies have shown the importance of dietary fibers for the growth of beneficial microbes [69,70,71,72].

Unfortunately, the questionnaire was not specific enough, nor was the sample size large enough, to allow for more in-depth evaluation of the effect of these two factors (probiotic and diet). Only 9 out of 55 participants had a diet low in processed food, which made it difficult to investigate the effect of processed food on bacteria and fungi. 

Nevertheless, the *p*-values of fungi taxa abundance were greatly affected by adding probiotic intake to the regression model (Appendix A Table A1). As the questionnaire did not specifically ask for which type of probiotic participants were taking, the assumption is that they used bacterial probiotics, as most probiotics sold over the counter are bacterial. Together, these data suggest, probably, that there is an interaction between bacteria and fungi that influences fungal abundance. Indeed, bacteria and fungi have been shown to interact in various ways, including cell contact, quorum sensing, pH changes and use of metabolites by-products [73].

As an increase in the abundance of *S. cereviciae* was noted in both GID and NDD groups, it is important to mention that this species is often named *S. boulardii*, a probiotic, that has a beneficial effect on several strains of enteropathogenic bacteria [32,74,75,76]. The present data question again whether *cereviciae* and *boulardii* are the same species [76]. 

### 4.4. Effect of Early Antibiotic Exposure

Slob et al. [35] showed in a cohort of 27,781 twins that early exposure to antibiotics significantly increased the risk of developing ADHD and ASD. As our previous study also showed a significant association between early exposure to recurrent courses of antibiotics developing an NDD [40], we decided to explore this question further and run both an independent t-test and a regression model to investigate the taxa that could be affected by early antibiotic exposure. While the sample size is relatively small, this preliminary exploration aims principally at drawing future directions of investigations. One of the genera that were affected is *Prevotella*. Interestingly, Figure 1A suggests a possible increase in NDD of the relative mean abundance of *Prevotella*. However, this is not significant with either of the models, even when using relative abundance. Nevertheless, the regression analysis using relative abundance and all covariates—including diet and probiotics—for the NDD group, showed a significant decrease in *Prevotella* in NDD participants who were exposed to recurrent courses of antibiotics before 3 years. Our limited sample size might explain why *Prevotella* did not show up in the significantly decreased genera in NDD, as shown by a high number of studies [77,78,79,80,81].

Results also suggest that early ABX exposure is associated with greater fungal dysbiosis than bacterial dysbiosis, which again suggests the existence of essential interactions between bacteria and fungi kingdoms. Indeed, studies show that antibiotics, although directly targeting bacteria, by creating an imbalance in bacteriome, can lead to fungal overgrowth of certain species that might be later involved in the production of immune response modulators [36,40,82].

The effect of antibiotics on gut microbiota depends on the type of antibiotic, timing and duration of consumption, and microbiome modulatory factors such as age, travel, underlying illness, antibiotic resistance pattern and diet. The administration of antibiotics during childhood usually consists of short courses of relatively narrow spectrum agents for respiratory tract and oropharyngeal infections [83]. The abundance of *Clostridiales* and *Ruminococcus* was reduced as a result of postnatal exposure to antibiotics. However, the exposed and non-exposed groups had a similar overall number of species and diversity after 1 year of life [84]. This effect can be magnified due to the excessive use of antibiotics or underlying gastrointestinal conditions [84]. Thus, antibiotics, diet and environment may substantially impact the development of the gut microbiome of the developing child. Therefore, antibiotics consumption may indirectly modulate the intestinal immune homeostasis by shifting the structure of the gut microbiota [85].

### 4.5. Important Taxa to Explore Further

The Firmicutes/Bacteroidetes (F/B) ratio is believed to play an important role in maintaining gut homeostasis (Stojanov et al., 2020). In fact, studies have shown an increase in this ratio. Contradicting results have been published earlier, some showing a decrease in this ratio due to a decrease of Bacteroidetes in ASD [86,87,88,89] and others showing an increase [90]. In GID, Firmicutes, which is normally the most dominant phylum in the microbiota of healthy guts, was observed at significantly lower levels, leading to an observed decrease in the F/B ratio [14,16]. In this study, the ratio of Firmicutes/Bacteroidetes was significantly decreased in NDD compared to CTRL (*p* = 0.045), and this was due to a decrease in Firmicutes. Interestingly, this same ratio in GID is significantly different from neither CTRL (*p*-value 3 = 0.402) nor NDD groups (*p*-value 3 = 0.87, data not shown), supporting the hypothesis that the “dysbiotic window” is extremely narrow and that small changes in bacteriome composition could result in association with different disorders. Many studies have explored the F/B ratio as acetate and propionate are mainly produced by Bacteroidetes, whereas Firmicutes contribute primarily to the production of butyrate [91,92,93]. Acetate, propionate and butyrate are short-chain fatty acids that have been shown to be essential for the development the immune system as well as neurodevelopment [64,91,92,93,94]. 

While the focus is often made on the F/B ratio, a change in the F/B ratio is often the result of concomitant changes in the levels of other phyla like Proteobacteria and Actinobacteria. Observing ratios can inform of the general dynamics between phyla. Therefore, the R package was used to investigate changes of all possible ratios at all taxa levels to explore the significant change in any other ratio. Table 1 shows only the significant ratio changes at the phylum level. While these ratios were not previously described, they are worth mentioning because of their significance. We noticed indeed a significant decrease in the Firmicutes/Actinobacteria ratio in NDD compared to both CTRL and GID, and this is due to an increase in Actinobacteria and a decrease in Firmicutes in the NDD group (data not shown).

Indeed, Actinobacteria, despite their lower abundance, also play an important role in the maintenance of gut homeostasis [95], and this is confirmed by studies on the probiotic role of *Bifidobacteria*, which is a class of Actinobacteria. Interestingly, the TM7 phylum or Saccharibacteria have been shown to be an obligate epibiont parasite of diverse Actinobacteria [96], and changes in TM7 might be due to changes in Actinobacteria. In the GID group, the ratios involving the Saccharibacteria TM7 are decreased: TM7/Actinobacteria (*p* = 0.041) and TM7/Firmicutes (*p* = 0.044). A study has investigated the association of TM7 with IBD and noted a genetic alteration for an antibiotic resistance gene of TM7 species in IBD participants [41]. 

Other taxa levels have shown significant changes in abundance and have also been mentioned by other studies, such as the orders of Enterobacteriales [86] and RF32 [97] and the family of *Enterobacteriaceae* and *Butyricicoccus* [86]. *Butyricicoccus pullicaecorum* is a butyrate-producing bacterium that has been shown by other studies to be significantly decreased in NDD compared to CTRL [98]. However, this current study shows that it is significantly increased in NDD compared to CTRL. Interestingly, a recent exploration of the gut microbiome of autistic infants in various age groups also found a higher relative abundance of *Butyricicoccus pullicaecorum* at 3 years old [55]. This observation emphasizes the importance of considering all factors that might influence the gut microbiome composition in statistical analyses, including age, and other factors mentioned [55]. *Coprococcus* is butyric acid-producing bacterium that has been shown to have a protective role. Its decrease in NDD shown in this present study has also been observed by a number of other studies [81,90,99].

Among the 28 fungal genera that significantly changed, *Saccharomycces* and its species *Saccharomycces cereviciae* are noted as one of the most significantly changed genus and species with an increased abundance in NDD (Table A1). While several studies have confirmed the same [33], some others have shown that the probiotic yeast *Saccharomyces boulardii* efficiently reduced anxiety-related symptoms in ASD [32,100,101]. Some studies consider S. *boulardii* as a strain of S. *cereviciae* [76], which would contradict our data. This raises again the question of whether S. *boulardii* is a distinct species from S. *cereviciae* [32,76].

### 4.6. Importance of the Gut Microbiome in Immunity Development

The human gut bacteriome contributes to the health of the host by producing short-chain fatty acids (SCFAs) such as butyrate, propionate and acetate. These are considered as a major source of energy for the intestinal epithelia in addition to their role in enhancing the integrity of the mucosal barrier [92,102]. With the decreased bacterial biodiversity observed in both GID and NDD, lower amounts of these SCFAs should be expected in future metabolomics explorations.

Moreover, the gut microbiota, including fungi, plays an essential role in modulating both the innate and adaptive arms of the immune response through various mechanisms [103,104,105,106]. by downregulating the secretion of the pro-inflammatory cytokines [107] and increasing levels of inflammatory markers such as lactoferrin are expected to be found in these groups.

Therefore, it is not surprising that auto-immunity is often mentioned to explain IBD, celiac disease and also ASD or ADHD [108,109]

Further metabolomics explorations are needed to determine the presence of immune alterations in NDD and GID and whether those can be reversible by targeting bacterial and/or the fungal gut dysbiosis.

## 5. Conclusions

In recent years, the gut microbiota has been considered as an important actor of the gut–brain axis, making it possible to take new steps in understanding neurological diseases and neurodevelopmental disorders as well as gastrointestinal disorders. While most studies, including large-scale projects such as the Human Microbiome project, were exclusively focusing on the bacteriome, the mycobiome was neglected. In this study, we unravel how, unlike for bacterial dysbiosis, the fungal dysbiosis in NDD is such that its diversity is increased while the abundance of specific taxa is decreased. The opposite pattern was observed with bacterial dysbiosis in NDD, with a decrease in alpha diversity and an increase in abundance of a high number of taxa.

**Figure 4:**
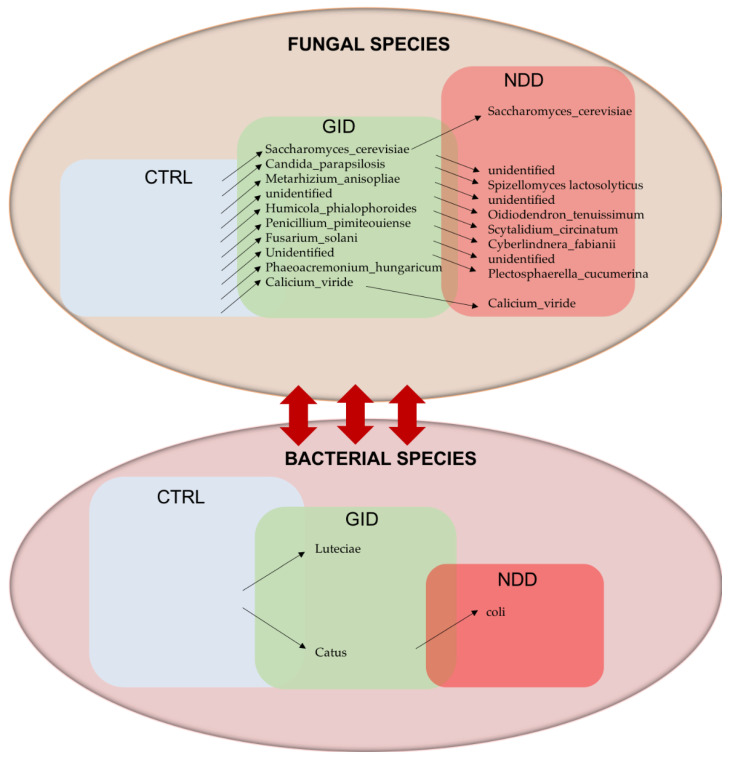
Fungal species as key players in GID and NDD. Arrows up show the increasing abundance of species, and arrows down show the decreasing abundance of species between two consecutive boxes. Height of the boxes CTRL, GID and NDD simulates the increasing alpha diversity for fungi and the decreasing alpha diversity for bacteria. The double arrow shows the interaction between bacteria and fungi.

While the GID group elicits only a small number of significant differences in bacterial abundances compared to CTRL and compared to NDD, the mycobiome seem to be the component that differentiates the GID and the NDD group. We identified 10 fungal species that increase in GID compared to CTRL and 8 other species that are increased in GID compared to NDD. Our model in Figure 4 summarizes these findings.

Interestingly, probiotic intake, diet and antibiotic exposure had a greater effect on fungal abundances than bacterial abundances, suggesting the presence of strong interactions between both kingdoms. These interactions will be key in further understanding the pathogenesis of various diseases of the gut–brain axis.

## Figures and Tables

**Table 1:** Characteristics of the studied cohort.

	CTRL	NDD	GID	TOTAL
Gender *N* (%)				
Male	10 (37)	11 (73)	5 (38)	26 (47)
Female	17 (63)	4 (27)	8 (62)	29 (52)
Total	27 (100)	15 (100)	13 (100)	55 (100)
Age *N* (%)				
3 to 18 years	12 (44)	14 (93)	3 (23)	29 (52)
19–60 years	15 (56)	1 (7)	10 (77)	26 (47)
Total	27 (100)	15 (100)	13 (100)	55 (100)
Ethnicity *N* (%)				
Middle Eastern	8 (30)	6 (40)	1 (8)	15 (27)
Caucasian	9 (33)	2 (13)	9 (69)	20 (11)
Indian	1 (4)	3 (20)	1 (8)	5 (9)
Asian	2 (7)	2 (13)	0 (0)	4 (7)
North African	2 (7)	0 (0)	2 (15)	4 (7)
Black African	1 (4)	1 (7)	0 (0)	2 (3)
Body mass index *N* (%)				
BMI ≥ 30	4 (15)	0 (0)	2 (15)	6 (11)
BMI < 30	22 (81)	11 (73)	11 (85)	44 (80)
Probiotic intake *N* (%)				
Regular intake	12 (44)	6 (40)	7 (54)	23 (42)
No regular intake	15 (56)	9 (60)	6 (46)	30 (55)
Diet in processed carbohydrates *N* (%)				
Medium to high	22 (81)	15 (100)	9 (69)	46 (84)
Low	5 (19)	0	4 (31)	9 (16)
Recurrent antibiotic use before 3Y *N* (%)				
Yes	7	10	6	23 (42)
No	20	5	5	30 (55)

**Table 2:** Significant phyla ratio changes among neurodevelopmental disorder (NDD) and gastrointestinal disorder (GID) groups.

	Phyla Ratio			Basic Covariates + Probiotics and Diet			
			DF	R-Squared	Beta	SEbeta	*p*-Value
NDD compared to CTRL	Proteobacteria/Cyanobacteria	↑	19	0.222	3413.585	1182.735	0.009
	Firmicutes/Actinobacteria	↓	29	0.253	−170.525	74.769	0.030
	Firmicutes/Bacteroidetes	↓	29	0.094	−0.721	0.345	0.045
	Proteobacteria/Verrucomicrobia	↑	27	0.179	848.553	404.793	0.046
GID compared to CTRL	Firmicutes/Verrucomicrobia	↑	28	0.182	9628.261	3295.069	0.007
	Cyanobacteria/Verrucomicrobia	↑	28	0.159	7.397	2.630	0.009
	Actinobacteria/Verrucomicrobia	↑	28	0.030	1278.024	576.498	0.035
	TM7/Actinobacteria	↓	31	0.113	−0.009	0.004	0.041
	TM7/Firmicutes	↓	31	0.066	0.000	0.000	0.044
NDD compared to GID	TM7/Actinobacteria	↓	42	0.125	−0.008	0.003	0.018
	Firmicutes/Verrucomicrobia	↑	38	0.109	6035.704	2508.904	0.021
	Proteobacteria/Verrucomicrobia	↑	38	0.193	756.313	320.375	0.023
	Firmicutes/Actinobacteria	↓	42	0.229	−106.975	49.164	0.035
	Cyanobacteria/Verrucomicrobia	↑	38	0.070	4.239	1.995	0.040

Arrows show the increase or decrease of the specified ratios in the first group compared to the second.

**Table 3:** Significant absolute abundances of bacterial taxa in NDD compared to CTRL.

Level		Name			Basic Covariates			Adding Probiotics	Adding Diet	Adding Probiotics and Diet
			DF	R-Squared	Beta	SEbeta	*p*-Value	*p*-Value 1	*p*-Value 2	*p*-Value 3
Order	↑	Enterobacteriales	32	0.170983	1.43611	0.447029	0.002996	0.002745	0.004335	0.003967
	↑	RF32	32	0.100373	0.257666	0.097869	0.012931	0.017538	0.017723	0.023618
Family	↑	*Enterobacteriaceae*	32	0.170983	1.43611	0.447029	0.002996	0.002745	0.004335	0.012903
	↑	*Enterococcaceae*	32	0.142559	1.225977	0.450581	0.010441	0.00933	0.014408	NS
Genus	↑	*SMB53*	32	0.298415	0.75418	0.21188	0.001185	0.001722	0.001206	0.001785
	↑	*Escherichia*	32	0.122884	1.231856	0.429424	0.007242	0.008244	0.009616	0.010944
	↑	*Clostridium*	32	0.193332	1.244652	0.434901	0.007365	0.009685	0.010454	0.013531
	↑	*Butyricicoccus*	32	0.137802	1.193238	0.431852	0.009413	0.009851	0.01305	0.013604
	↑	*Enterococcus*	32	0.142559	1.225977	0.450581	0.010441	0.00933	0.014408	0.012903
	↑	*Lactococcus*	32	0.138711	0.12109	0.049673	0.020514	0.030686	0.019488	0.02914
	↑	*Veillonella*	32	0.066232	1.126486	0.464704	0.021175	0.029099	0.029006	0.039101
	↓	*Coprococcus*	32	0.10669	−0.60313	0.29107	0.046392	0.046974	NS	0.003967
Species	↑	*coli*	32	0.122884	1.231856	0.429424	0.007242	0.008244	0.009616	0.010944
	↑	*pullicaecorum*	32	0.137802	1.193238	0.431852	0.009413	0.009851	0.01305	0.013604
	↑	*dispar*	32	0.050691	1.005875	0.471401	0.040622	NS	NS	NS
	↑	*bifidum*	32	0.034055	0.899072	0.439749	0.049203	NS	NS	NS

Arrows show the increase or decrease of mean absolute abundance in the GID group compared to CTRL.

**Table 4:** Significant relative abundances of bacterial taxa in NDD compared to CTRL.

Level		Name	Basic Covariates					Adding Probiotics	Adding Diet	Adding Probiotics and Diet
			DF	R-Squared	Beta	SEbeta	*p*-Value	*p*-Value 1	*p*-Value 2	*p*-Value 3
Phylum	↓	Firmicutes	32	0.077031	−0.95357	0.366404	0.013907	0.014174	0.007765	0.007917
Order	↑	Enterobacteriales	32	0.168988	1.447609	0.445448	0.002717	0.002453	0.003935	0.003554
	↓	Clostridiales	32	0.115446	−1.03299	0.356563	0.006743	0.007301	0.003770	0.004081
	↑	RF32	32	0.052137	0.185216	0.090321	0.048567	NS	NS	NS
Family	↑	*Enterobacteriaceae*	32	0.168988	1.447609	0.445448	0.002717	0.002453	0.003935	0.003554
	↑	*Brevibacteriaceae*	32	0.133259	1.249522	0.433006	0.006939	0.009815	0.008963	0.012486
	↑	*Enterococcaceae*	32	0.144581	1.225499	0.447687	0.010027	0.009193	0.013925	0.012765
	↓	*Mogibacteriaceae*	32	0.065234	−0.80553	0.385277	0.044577	NS	NS	NS
	↑	*Corynebacteriaceae*	32	0.02451	−0.5357	0.3331	NS	NS	NS	0.041973
Genus	↑	*SMB53*	32	0.225957	0.647016	0.220494	0.006137	0.008254	0.006756	0.009169
	↑	*Brevibacterium*	32	0.133259	1.249522	0.433006	0.006939	0.009815	0.008963	0.012486
	↑	*Enterococcus*	32	0.144581	1.225499	0.447687	0.010027	0.009193	0.013925	0.012765
	↑	*Clostridium*	32	0.181039	1.147432	0.433232	0.012448	0.016721	0.017419	0.023002
	↑	*Escherichia*	32	0.091324	1.123838	0.436121	0.014785	0.017222	0.018506	0.021551
	↑	*Butyricicoccus*	32	0.111908	1.060691	0.413567	0.015219	0.016210	0.020536	0.021816
	↑	*Veillonella*	32	0.055591	1.079019	0.461371	0.025758	0.035185	0.035173	0.047124
	↓	*Coprococcus*	32	0.112285	−0.70915	0.316395	0.032068	0.032769	0.036046	0.036777
	↓	*Odoribacter*	32	0.1039	−0.880	0.4329	NS	0.049563	0.047686	0.047292
Species	↑	*aureum*	32	0.133259	1.249522	0.433006	0.006939	0.009815	0.008963	0.012486
	↑	*coli*	32	0.091324	1.123838	0.436121	0.014785	0.017222	0.018506	0.021551
	↑	*pullicaecorum*	32	0.111908	1.060691	0.413567	0.015219	0.016210	0.020536	0.021816
	↓	*dolichum*	32	0.102217	-0.11802	0.054746	0.038727	NS	0.043557	NS
	↑	*dispar*	32	0.045073	0.989858	0.465437	0.041250	NS	NS	NS
	↑	*faecis*	32	0.031296	0.477281	0.229260	0.045439	0.037505	NS	0.045605
	↑	*bifidum*	32	0.038356	0.897201	0.434263	0.047000	NS	NS	NS

Arrows show the increase or decrease of mean relative abundance in the NDD group compared to CTRL. Arrows show the increase or decrease of mean absolute abundance in GID group compared to CTRL.

**Table 5:** Significant relative abundances of bacterial taxa in GID compared to CTRL.

Level		Name	Basic Covariates					Adding Probiotics	Adding Diet	Adding Probiotics and Diet
			DF	R-Squared	Beta	Sebeta	*p*-Value	*p*-Value 1	*p*-Value 2	*p*-Value 3
Family	↓	*Odoribacteraceae*	34	0.04502157	−0.7451711	0.35897333	0.04554079	NS	0.04884609	NS
Genus	↑	*Anaerotruncus*	34	0.18021437	0.54957278	0.1741761	0.00334826	0.00463763	0.00201844	0.00288391
	↓	*Butyricimonas*	34	0.14848867	−0.6716181	0.27245144	0.01890498	0.02584356	0.02047021	0.02777944
Species	↓	*catus*	34	0.082374	−0.8869353	0.35892387	0.01863917	0.01932181	0.01999275	0.0205811
	↑	*luteciae*	34	0.07544368	0.22614282	0.11062371	0.04873506	NS	NS	NS

Arrows show the increase or decrease of mean relative abundance in GID group compared to CTRL.

**Table 6:** Significant absolute abundances of bacterial taxa in NDD compared to GID.

Level		Name	Basic Covariates					Adding Probiotics	Adding Diet	Adding Probiotics and Diet
			DF	R-Squared	Beta	Sebeta	*p*-Value	*p*-Value 1	*p*-Value 2	*p*-Value 3
no significant difference in any taxa abundances between NDD and GID at Phylum, Class, Family, Order levels across all 4 models										
Genus	↑	*SMB53*	45	0.0566	0.6860	0.2891	0.0220	0.0297	0.0234	0.0317
	↓	*Odoribacter*	45	0.1602	−0.5429	0.2629	0.0447	0.0417	0.0473	0.0441
	↑	*Anaerotruncus*	45	0.0435	0.5814	0.2888	0.0501	NS	0.0534	NS
	↑	*Escherichia*	43	0.0048	0.6123	0.3051	NS	0.0510	0.0444	0.0548
Species	↑	*coli*	45	0.0247	0.6246	0.2975	0.0414	0.0510	0.0444	0.0548

**Table 7:** Significant absolute abundance in NDD compared to CTRL at the species level.

Increased Absoulte Abundance	
Species	*p*-Value Basic Covariates + Probiotics + Diet
*Unidentified*/k__Fungi;p__Ascomycota;c__Leotiomycetes;o__unidentified;f__unidentified;g__unidentified;s__unidentified;	0.0499
*Peziza_nivalis*	0.0260
*Chaetomium_erectum*	0.0176
*Melanophyllum_haematospermum*	0.0190
*Unidentified*/k__Fungi;p__Ascomycota;c__Dothideomycetes;o__Capnodiales;f__Mycosphaerellaceae;g__unidentified;s__unidentified;	0.0247
Arnium_arizonense	0.0370
*Unidentified*/k__Fungi;p__Ascomycota;c__Sordariomycetes;o__Microascales;f__Halosphaeriaceae;g__unidentified;s__unidentified;	0.0246
*Phialophora_mustea*	0.0404
*Unidentified*/k__Fungi;p__Ascomycota;c__Eurotiomycetes;o__Onygenales;f__unidentified;g__unidentified;s__unidentified;	0.0266
*Exophiala_dermatitidis*	0.0413
*Dioszegia_fristingensis*	0.0413
*Pyrenochaeta_keratinophila*	0.0413
*Exophiala_oligosperma*	0.0413
*Alternaria_hungarica*	0.0413
*Peziza_buxea*	0.0413
*Tetragoniomyces_uliginosus*	0.0413
*Unidentified*/k__Fungi;p__Basidiomycota;c__Agaricomycetes;o__Agaricales;f__Omphalotaceae;g__Marasmiellus;s__unidentified;	0.0413
*Unidentified*/k__Fungi;p__Ascomycota;c__Pezizomycetes;o__Pezizales;f__Pyronemataceae;g__Trichophaea;s__unidentified;	0.0413
*Exophiala_phaeomuriformis*	0.0413
*Unidentified*/k__Fungi;p__Ascomycota;c__Eurotiomycetes;o__Chaetothyriales;f__Herpotrichiellaceae;g__Phialophora;s__unidentified;	0.0413
*Aspergillus_ochraceus*	0.0413
*Mortierella_zonata*	0.0413
*Unidentified*/k__Fungi;p__Ascomycota;c__Dothideomycetes;o__Pleosporales;f__Leptosphaeriaceae;g__Leptosphaeria;s__unidentified;	0.0413
*Microascus_albonigrescens*	0.0413
*Coniophora_olivacea*	0.0420
*Unidentified*/k__Fungi;p__Ascomycota;c__Dothideomycetes;o__Pleosporales;f__Phaeosphaeriaceae;g__Chaetosphaeronema;s__unidentified;	0.0379
**Decreased Absolute Abundance**	
**Species**	***p*** **-Value Basic Covariates + Probiotics + Diet**
*Betamyces_americae-meridionalis*	0.0510
*Unidentified*/k__Fungi;p__Chytridiomycota;c__Rhizophydiomycetes;o__Rhizophydiales;f__unidentified;g__unidentified;s__unidentified;	0.0183
*Spizellomyces_dolichospermus*	0.0442
*Unidentified*/k__Fungi;p__Ascomycota;c__Leotiomycetes;o__Helotiales;f__unidentified;g__unidentified;s__unidentified;	0.0160
*Unidentified*/k__Fungi;p__Basidiomycota;c__Agaricomycetes;o__Auriculariales;f__unidentified;g__unidentified;s__unidentified;	0.0124
*Mortierella_amoeboidea*	0.0549
*Unide*ntified/k__Fungi;p__Mortierellomycota;c__Mortierellomycetes;o__Mortierellales;f__Mortierellaceae;g__Mortierella;s__unidentified;	0.0140
*Unidentified*/k__Fungi;p__Ascomycota;c__Sordariomycetes;o__Branch06;f__unidentified;g__unidentified;s__unidentified;	0.0428
*Unidentified/*k__Fungi;p__Ascomycota;c__unidentified;o__unidentified;f__unidentified;g__unidentified;s__unidentified;	0.0433
*Plectosphaerella_cucumerina*	0.0531

**Table 8:** Significant changes in Fungal abundance in NDD compared to GID.

				DF	R-Squared	Beta	SEbeta	*p* Value 3
Phylum	Rozellomycota	k__Fungi;p__Rozellomycota;	↓	21	0.1222	−0.7622	0.3547	0.0434
Class	Wallemiomycetes	k__Fungi;p__Basidiomycota;c__Wallemiomycetes;	↓	21	0.3717	−0.8819	0.2930	0.0067
	Spizellomycetes	k__Fungi;p__Chytridiomycota;c__Spizellomycetes;	↓	21	0.1580	−0.9972	0.3424	0.0083
	Agaricomycetes	k__Fungi;p__Basidiomycota;c__Agaricomycetes;	↓	21	0.3393	−0.8713	0.3415	0.0186
	Orbiliomycetes	k__Fungi;p__Ascomycota;c__Orbiliomycetes;	↑	21	0.0222	0.7902	0.3669	0.0431
Order	Wallemiales	k__Fungi;p__Basidiomycota;c__Wallemiomycetes;o__Wallemiales;	↓	21	0.3717	−0.8819	0.2930	0.0067
	Spizellomycetales	k__Fungi;p__Chytridiomycota;c__Spizellomycetes;o__Spizellomycetales;	↓	21	0.1580	−0.9972	0.3424	0.0083
	Branch06	k__Fungi;p__Ascomycota;c__Sordariomycetes;o__Branch06;	↓	21	0.1625	−0.8069	0.3433	0.0286
	Orbiliales	k__Fungi;p__Ascomycota;c__Orbiliomycetes;o__Orbiliales;	↑	21	−0.0004	0.8219	0.3895	0.0470
Family	*Wallemiales_fam_Incertae_sedis*	k__Fungi;p__Basidiomycota;c__Wallemiomycetes;o__Wallemiales;f__Wallemiales_fam_Incertae_sedis;	↓	21	0.3717	−0.8819	0.2930	0.0067
	*Spizellomycetaceae*	k__Fungi;p__Chytridiomycota;c__Spizellomycetes;o__Spizellomycetales;f__Spizellomycetaceae;	↓	21	0.0651	−0.9407	0.3653	0.0177
	*unidentified*	k__Fungi;p__Ascomycota;c__Sordariomycetes;o__Branch06;f__unidentified;	↓	21	0.1625	−0.8069	0.3433	0.0286
	*Phaffomycetaceae*	k__Fungi;p__Ascomycota;c__Saccharomycetes;o__Saccharomycetales;f__Phaffomycetaceae;	↓	21	0.3319	−0.7243	0.3141	0.0314
	*Leotiomycetes_fam_Incertae_sedis*	k__Fungi;p__Ascomycota;c__Leotiomycetes;o__Leotiomycetes_ord_Incertae_sedis;f__Leotiomycetes_fam_Incertae_sedis;	↓	21	0.1523	−0.7956	0.3477	0.0326
Genus	*Wallemia*	k__Fungi;p__Basidiomycota;c__Wallemiomycetes;o__Wallemiales;f__Wallemiales_fam_Incertae_sedis;g__Wallemia;	↓	21	0.3717	−0.8819	0.2930	0.0067
	*Saccharomyces*	k__Fungi;p__Ascomycota;c__Saccharomycetes;o__Saccharomycetales;f__Saccharomycetaceae;g__Saccharomyces;	↑	21	0.2563	0.9880	0.3354	0.0077
	*Spizellomyces*	k__Fungi;p__Chytridiomycota;c__Spizellomycetes;o__Spizellomycetales;f__Spizellomycetaceae;g__Spizellomyces;	↓	21	0.0691	−0.9486	0.3713	0.0185
	*unidentified*	k__Fungi;p__Ascomycota;c__Sordariomycetes;o__Branch06;f__unidentified;g__unidentified;	↓	21	0.1625	−0.8069	0.3433	0.0286
	*Cyberlindnera*	k__Fungi;p__Ascomycota;c__Saccharomycetes;o__Saccharomycetales;f__Phaffomycetaceae;g__Cyberlindnera;	↓	21	0.2069	−0.8408	0.3647	0.0314
	*Scytalidium*	k__Fungi;p__Ascomycota;c__Leotiomycetes;o__Leotiomycetes_ord_Incertae_sedis;f__Leotiomycetes_fam_Incertae_sedis;g__Scytalidium;	↓	21	0.1523	−0.7956	0.3477	0.0326
	*Plectosphaerella*	k__Fungi;p__Ascomycota;c__Sordariomycetes;o__Glomerellales;f__Plectosphaerellaceae;g__Plectosphaerella;	↓	21	0.1150	−0.5678	0.2598	0.0403
	*unidentified*	k__Fungi;p__Ascomycota;c__Dothideomycetes;o__Dothideomycetidae_ord_Incertae_sedis;f__Eremomycetaceae;g__unidentified;	↑	21	0.0949	0.7849	0.3637	0.0426
Species	*unidentified*	k__Fungi;p__Basidiomycota;c__Wallemiomycetes;o__Wallemiales;f__Wallemiales_fam_Incertae_sedis;g__Wallemia;s__unidentified;	↓	21	0.4107	−0.9420	0.2880	0.0036
	*Saccharomyces_cerevisiae*	k__Fungi;p__Ascomycota;c__Saccharomycetes;o__Saccharomycetales;f__Saccharomycetaceae;g__Saccharomyces;s__Saccharomyces_cerevisiae;	↑	21	0.2563	0.9880	0.3354	0.0077
	*Spizellomyces_lactosolyticus*	k__Fungi;p__Chytridiomycota;c__Spizellomycetes;o__Spizellomycetales;f__Spizellomycetaceae;g__Spizellomyces;s__Spizellomyces_lactosolyticus;	↓	21	0.0336	−0.9039	0.3694	0.0233
	*unidentified*	k__Fungi;p__Ascomycota;c__Sordariomycetes;o__Branch06;f__unidentified;g__unidentified;s__unidentified;	↓	21	0.1625	−0.8069	0.3433	0.0286
	*Oidiodendron_tenuissimum*	k__Fungi;p__Ascomycota;c__Leotiomycetes;o__Helotiales;f__Myxotrichaceae;g__Oidiodendron;s__Oidiodendron_tenuissimum;	↓	21	0.2239	−0.8286	0.3600	0.0317
	*Scytalidium_circinatum*	k__Fungi;p__Ascomycota;c__Leotiomycetes;o__Leotiomycetes_ord_Incertae_sedis;f__Leotiomycetes_fam_Incertae_sedis;g__Scytalidium;s__Scytalidium_circinatum;	↓	21	0.1523	−0.7956	0.3477	0.0326
	*Cyberlindnera_fabianii*	k__Fungi;p__Ascomycota;c__Saccharomycetes;o__Saccharomycetales;f__Phaffomycetaceae;g__Cyberlindnera;s__Cyberlindnera_fabianii;	↓	21	0.1991	−0.8316	0.3662	0.0338
	*Plectosphaerella_cucumerina*	k__Fungi;p__Ascomycota;c__Sordariomycetes;o__Glomerellales;f__Plectosphaerellaceae;g__Plectosphaerella;s__Plectosphaerella_cucumerina;	↓	21	0.1150	−0.5678	0.2598	0.0403
	*unidentified*	k__Fungi;p__Ascomycota;c__Dothideomycetes;o__Dothideomycetidae_ord_Incertae_sedis;f__Eremomycetaceae;g__unidentified;s__unidentified;	↑	21	0.0949	0.7849	0.3637	0.0426
	*Calicium_viride*	k__Fungi;p__Ascomycota;c__Lecanoromycetes;o__Caliciales;f__Caliciaceae;g__Calicium;s__Calicium_viride;	↓	21	0.2079	−0.5845	0.2745	0.0452

**Table 9:** Fungi in GID compared to CTRL at the species level.

Increased in GID vs. CTRL					Decreased in GID vs. CTRL
Species	R-squared	beta	SEbeta	*p*-value basic covariates + probiotics + diet	no species significantly decreased in GID compared to CTRL
*Saccharomyces_cerevisiae*	0.27665113	1.09894673	0.34372426	0.01138158	
*Candida_parapsilosis*	0.28455775	1.05552026	0.37516274	0.01592194	
*Metarhizium_anisopliae*	0.2610366	0.14201073	0.05109858	0.02205624	
*unidentified*	0.21517888	0.9223066	0.37481416	0.0260362	
*Humicola_phialophoroides*	0.10755173	0.81109936	0.35117492	0.04047386	
*Penicillium_pimiteouiense*	0.17719919	0.85081527	0.36915659	0.03106863	
*Fusarium_solani*	0.17912126	0.90662454	0.41162482	0.04566077	
*unidentified*	0.46101634	−0.9334353	0.31142748	0.00943343	
*Phaeoacremonium_hungaricum*	0.19271259	0.48732341	0.22192725	0.04648654	
*Calicium_viride*	0.16255788	−0.6647188	0.31299161	0.04865476	

**Table 10:** Significant *p*-values using the *t*-test for +ABX vs. -ABX for bacteria and fungi at all taxa levels.

T-TEST ABX VS. NON-ABX_BACTERIA								
	Name	Statistic	df	*p*-Value	CI	CI	Mean of +abx	Mean of −abx
Phylum	TM7	2.0450	26	0.0509	−0.0018	0.8286	0.5465	0.1331
Class	TM7-3	2.0450	26	0.0509	−0.0018	0.8286	0.5465	0.1331
Family	*Succinivibrionaceae*	−2.3054	30	0.0281	−0.0713	−0.0043	−0.0512	−0.0134
Genus	*Butyricimonas*	−3.3256	38	0.0020	−0.8139	−0.1979	−0.2551	0.2508
	*Dorea*	−2.4678	49	0.0172	−0.3432	−0.0351	−0.2667	−0.0776
	*Eggerthella*	2.4743	21	0.0219	0.0794	0.9150	0.3092	−0.1880
	*Succinivibrio*	−2.3054	30	0.0281	−0.0713	−0.0043	−0.0512	−0.0134
Species	*eggerthii*	−2.3689	49	0.0218	−0.1606	−0.0132	−0.0693	0.0176
	*lenta*	2.4743	21	0.0219	0.0794	0.9150	0.3092	−0.1880
**T-TEST ABX VS. NON-ABX_FUNGI**								
	**Name**	**Statistic**	**df**	***p*-Value**	**CI**	**CI**	**Mean of +abx**	**Mean of −abx**
Phylum	Neocallimastigomycota	−2.4086	27	0.0231	−0.2759	−0.0221	−0.1031	0.0459
Class	Ascomycota_cls_Incertae_sedis	−2.5506	28	0.0165	−0.7215	−0.0787	−0.0487	0.3514
	Neocallimastigomycetes	−2.4086	27	0.0231	−0.2759	−0.0221	−0.1031	0.0459
Order	Ascomycota_ord_Incertae_sedis	−2.5506	28	0.0165	−0.7215	−0.0787	−0.0487	0.3514
	Neocallimastigales	−2.4086	27	0.0231	−0.2759	−0.0221	−0.1031	0.0459
	Coniochaetales	−2.3188	25	0.0287	−0.4096	−0.0244	−0.0277	0.1893
	Caliciales	−2.0308	26	0.0526	−0.4670	0.0029	0.0024	0.2345
Family	*Ascomycota_fam_Incertae_sedis*	−2.5506	28	0.0165	−0.7215	−0.0787	−0.0487	0.3514
	*unidentified*/k__Fungi;p__Mortierellomycota;c__Mortierellomycetes;o__Mortierellales;f__*unidentified*;	2.7003	13	0.0184	0.1502	1.3624	0.6390	−0.1173
	*unidentified*/k__Fungi;p__Ascomycota;c__Dothideomycetes;o__Pleosporales;f__*unidentified*;	−2.4067	29	0.0226	−1.0320	−0.0841	−0.1434	0.4147
	*Neocallimastigaceae*	−2.4086	27	0.0231	−0.2759	−0.0221	−0.1031	0.0459
	*Cortinariaceae*	−2.2683	24	0.0326	−0.3772	−0.0178	−0.0234	0.1741
	*Caliciaceae*	−2.0308	26	0.0526	−0.4670	0.0029	0.0024	0.2345
Genus	*unidentified/*k__Fungi;p__Mortierellomycota;c__Mortierellomycetes;o__Mortierellales;f*__unidentified;g__unidentified*;	2.7003	13	0.0184	0.1502	1.3624	0.6390	−0.1173
	*unidentified*/k__Fungi;p__Ascomycota;c__Dothideomycetes;o__Pleosporales;*f__unidentified;g__unidentified*;	−2.4067	29	0.0226	−1.0320	−0.0841	−0.1434	0.4147
	*unidentified*/k__Fungi;p__Neocallimastigomycota;c__Neocallimastigomycetes;o__Neocallimastigales;*f__Neocallimastigaceae;g__unidentified*;	−2.4086	27	0.0231	−0.2759	−0.0221	−0.1031	0.0459
	*unidentified*/k__Fungi;p__Ascomycota;c__Sordariomycetes;o__Glomerellales;*f__Plectosphaerellaceae;g__unidentified*;	2.6718	9	0.0254	0.1292	1.5437	0.8394	0.0029
	*unidentified*/k__Fungi;p__Ascomycota;c__Pezizomycetes;o__Pezizales;*f__Pyronemataceae;g__unidentified*;	−2.2488	28	0.0325	−0.4571	−0.0215	−0.0731	0.1662
	*Calicium*	−2.1900	28	0.0369	−0.5191	−0.0175	−0.0296	0.2387
	*Neurospora*	−2.1516	21	0.0432	−0.6826	−0.0116	0.0000	0.3471
	*unidentified*/k__Fungi;p__Ascomycota;c__Dothideomycetes;o__Pleosporales;*f__Pleosporaceae;g__unidentified*;	−2.1190	22	0.0456	−0.3204	−0.0035	−0.0124	0.1495
	*Didymella*	−2.0777	28	0.0469	−0.7601	−0.0056	−0.0625	0.3203
	*unidentified*/k__Fungi;p__Ascomycota;c__Sordariomycetes;o__Sordariales;*f__Sordariaceae;g__unidentified*;	−2.0729	21	0.0507	−0.6871	0.0011	0.0000	0.3430
Specie	*unidentified*/k__Fungi;p__Mortierellomycota;c__Mortierellomycetes;o__Mortierellales;*f__unidentified;g__unidentified;s__unidentified*;	2.7003	13	0.0184	0.1502	1.3624	0.6390	−0.1173
	*unidentified*/k__Fungi;p__Ascomycota;c__Dothideomycetes;o__Pleosporales;*f__unidentified;g__unidentified;s__unidentified*;	−2.4067	29	0.0226	−1.0320	−0.0841	−0.1434	0.4147
	*unidentified*/k__Fungi;p__Neocallimastigomycota;c__Neocallimastigomycetes;o__Neocallimastigales;*f__Neocallimastigaceae;g__unidentified;s__unidentified*;	−2.4086	27	0.0231	−0.2759	−0.0221	−0.1031	0.0459
	*unidentified*/k__Fungi;p__Ascomycota;c__Sordariomycetes;o__Glomerellales;*f__Plectosphaerellaceae;g__unidentified;s__unidentified*;	2.6718	9	0.0254	0.1292	1.5437	0.8394	0.0029
	*unidentified*/k__Fungi;p__Ascomycota;c__Pezizomycetes;o__Pezizales;*f__Pyronemataceae;g__unidentified;s__unidentified*;	−2.2488	28	0.0325	−0.4571	−0.0215	−0.0731	0.1662
	*Oidiodendron_truncatum*	−2.1978	20	0.0399	−0.6164	−0.0161	0.0007	0.3170
	*Neurospora_terricola*	−2.1516	21	0.0432	−0.6826	−0.0116	0.0000	0.3471
	*Rhodotorula_mucilaginosa*	−2.1079	27	0.0444	−0.3907	−0.0053	−0.0745	0.1235
	*unidentified*/k__Fungi;p__Ascomycota;c__Dothideomycetes;o__Pleosporales;f*__Pleosporaceae;g__unidentified;s__unidentified*;	−2.1190	22	0.0456	−0.3204	−0.0035	−0.0124	0.1495
	*Didymella_calidophila*	−2.0777	28	0.0469	−0.7601	−0.0056	−0.0625	0.3203
	*unidentified*/k__Fungi;p__Ascomycota;c__Sordariomycetes;o__Sordariales;f__Sordariaceae;g__unidentified;s__unidentified;	−2.0729	21	0.0507	−0.6871	0.0011	0.0000	0.3430
	*unidentified*/k__Fungi;p__Ascomycota;c__Sordariomycetes;o__Hypocreales;*f__Cordycipitaceae;g__Lecanicillium;s__unidentified*;	−2.0597	21	0.0520	−0.2603	0.0013	0.0000	0.1295

**Table 11:** Significant changes in bacterial and fungal absolute abundance with ABX exposure in NDD.

BACTERIA_NDD_ABX						
NAME		DF	R-Squared	Beta	SEbeta	*p*-Value
Family	*Campylobacteraceae*	29	0.1678	−0.1755	0.0740	0.0246
	*Corynebacteriaceae*	28	0.1823	−0.1587	0.0735	0.0395
Genus	*Rothia*	28	0.2196	0.3948	0.1658	0.0243
	*Campylobacter*	29	0.1678	−0.1755	0.0740	0.0246
	*WAL_1855D*	29	0.1869	−0.4043	0.1749	0.0281
	*Mogibacterium*	29	0.2957	0.3538	0.1532	0.0283
	*Butyricimonas*	28	0.1855	0.6542	0.2915	0.0329
	*Adlercreutzia*	29	0.1526	0.5718	0.2629	0.0380
	*Corynebacterium*	28	0.1823	−0.1587	0.0735	0.0395
	*Lactococcus*	30	0.2294	0.0998	0.0475	0.0440
Species	*zeae*	29	0.2868	0.0120	0.0043	0.0092
	*eggerthii*	30	0.0677	0.1520	0.0625	0.0213
**FUNGI_NDD_ABX**						
		**DF**	**R-Squared**	**Beta**	**SEbeta**	***p*-Value**
Phylum	Olpidiomycota	12	0.2202	−1.2000	0.4241	0.0152
Class	Olpidiomycetes	12	0.2225	−1.2098	0.4265	0.0150
Order	Leotiomycetes_ord_Incertae_sedis	12	0.3393	−1.4495	0.4912	0.0121
	Olpidiales	12	0.2225	−1.2098	0.4265	0.0150
	Chaetothyriales	12	0.2304	−0.3137	0.1328	0.0359
	Sebacinales	12	0.0227	−0.6656	0.3112	0.0537
Family	*Bolbitiaceae*	12	0.1570	−0.5405	0.1897	0.0146
	*Unidentified*/k__Fungi;p__Olpidiomycota;c__Olpidiomycetes;o__Olpidiales;f__unidentified;	12	0.1817	−0.9807	0.3936	0.0283
	*Bulleraceae*	13	0.3745	0.6050	0.2638	0.0391
	*Cyphellophoraceae*	12	0.4351	0.0220	0.0100	0.0485
Genus	*Spizellomyces*	11	0.6659	−0.8910	0.2156	0.0017
	*Wickerhamomyces*	13	0.5155	−1.2559	0.3417	0.0028
	*Mycothermus*	12	0.4477	−0.6108	0.1895	0.0073
	*Unidentified*/k__Fungi;p__Olpidiomycota;c__Olpidiomycetes;o__Olpidiales;f__unidentified;g__unidentified;	12	0.1817	−0.9807	0.3936	0.0283
	*Chaetosphaeria*	12	0.4210	0.4878	0.2078	0.0369
	*Unidentified*/ k__Fungi;p__Ascomycota;c__Sordariomycetes;o__Glomerellales;f__Plectosphaerellaceae;g__unidentified;	12	0.3705	−0.5830	0.2507	0.0384
	*Bullera*	13	0.3745	0.6050	0.2638	0.0391
	*Rhodotorula*	12	0.2511	0.3827	0.1657	0.0395
	*Torulaspora*	13	0.4224	0.5985	0.2622	0.0399
	*Cyphellophora*	12	0.4351	0.0220	0.0100	0.0485
	*Cercosporella*	13	0.0391	−0.4085	0.1930	0.0541
Specie	*Mycothermus_thermophilus*	12	0.4477	−0.6108	0.1895	0.0073
	*Unidentified*/k__Fungi;p__Olpidiomycota;c__Olpidiomycetes;o__Olpidiales;f__unidentified;g__unidentified;s__unidentified;	12	0.1817	−0.9807	0.3936	0.0283
	*Pichia_membranifaciens*	12	0.3877	0.3491	0.1427	0.0309
	*Chaetosphaeria_chloroconia*	12	0.4224	0.4856	0.2079	0.0376
	*Penicillium_pimiteouiense*	13	0.2521	−0.5084	0.2202	0.0380
	*Unidentified*/ k__Fungi;p__Ascomycota;c__Sordariomycetes;o__Glomerellales;f__Plectosphaerellaceae;g__unidentified;s__unidentified;	12	0.3705	−0.5830	0.2507	0.0384
	*Bullera_unica*	13	0.3744	0.6056	0.2640	0.0391
	*Torulaspora_delbrueckii*	13	0.4223	0.5982	0.2622	0.0400
	*Cutaneotrichosporon_cyanovorans*	12	0.4212	0.6892	0.3091	0.0457
	*Candida_glaebosa*	12	0.4241	−0.0525	0.0236	0.0461
	*Malassezia_sympodialis*	12	0.0533	−0.1744	0.0788	0.0471
	*Unidentified*/k__Fungi;p__Basidiomycota;c__Agaricomycetes;o__Agaricales;f__Amanitaceae;g__Amanita;s__unidentified;	13	0.1741	0.0428	0.0200	0.0519
	*Cercosporella_tinosporae*	13	0.0391	−0.4085	0.1930	0.0541

**Table 12:** Significant changes in bacterial and fungal absolute abundance with ABX exposure in GID.

BACTERIA_GID_ABX						
		DF	R-Squared	Beta	SEbeta	*p*-Value
Genus	*Atopobium*	28	0.0880	0.3233	0.1487	0.0383
	*Allobaculum*	29	0.0745	−0.0892	0.0436	0.0502
species	*eggerthii*	30	0.0494	0.1431	0.0625	0.0292
**FUNGI_GID_ABX**						
	**NAME**	**DF**	**R-Squared**	**Beta**	**SEbeta**	***p*-Value**
Phylum	Olpidiomycota	17	0.1176	−0.9239	0.3416	0.0150
	Neocallimastigomycota	17	0.2300	0.2260	0.0970	0.0325
Class	Olpidiomycetes	17	0.2028	−0.9728	0.3292	0.0089
	Neocallimastigomycetes	17	0.2300	0.2260	0.0970	0.0325
	Ascomycota_cls_Incertae_sedis	17	0.1328	0.6103	0.2704	0.0375
Order	Olpidiales	17	0.2028	−0.9728	0.3292	0.0089
	Neocallimastigales	17	0.2300	0.2260	0.0970	0.0325
	Ascomycota_ord_Incertae_sedis	17	0.1328	0.6103	0.2704	0.0375
Family	*Herpotrichiellaceae*	18	0.2303	−0.1517	0.0522	0.0094
	*Unidentified*/k__Fungi;p__Olpidiomycota;c__Olpidiomycetes;o__Olpidiales;f__unidentified;	17	0.1568	−0.8076	0.3105	0.0186
	*Debaryomycetaceae*	17	0.2249	−0.7173	0.2959	0.0268
	*Neocallimastigaceae*	17	0.2300	0.2260	0.0970	0.0325
	*Ascomycota_fam_Incertae_sedis*	17	0.1328	0.6103	0.2704	0.0375
	*Piskurozymaceae*	17	0.0240	0.0370	0.0178	0.0534
Genus	*Spizellomyces*	16	0.4395	−0.6295	0.1916	0.0047
	*Mycothermus*	17	0.3457	−0.6010	0.1907	0.0058
	*Wickerhamomyces*	17	0.3491	−0.9463	0.3095	0.0071
	*Neonectria*	18	0.1834	−0.6190	0.2156	0.0102
	*Unidentified*/k__Fungi;p__Olpidiomycota;c__Olpidiomycetes;o__Olpidiales;f__unidentified;g__unidentified;	17	0.1568	−0.8076	0.3105	0.0186
	*Vishniacozyma*	17	0.1886	−0.6668	0.2586	0.0195
	*Unidentified*/k__Fungi;p__Ascomycota;c__Sordariomycetes;o__Glomerellales;f__Plectosphaerellaceae;g__unidentified;	17	0.2170	−0.7604	0.3030	0.0225
	*Debaryomyces*	17	0.2666	−0.6988	0.2822	0.0241
	*Penicillium*	16	0.1926	−0.2792	0.1122	0.0242
	*Unidentified*/k__Fungi;p__Neocallimastigomycota;c__Neocallimastigomycetes;o__Neocallimastigales;f__Neocallimastigaceae;g__unidentified;	17	0.2300	0.2260	0.0970	0.0325
Specie	*Mycothermus*_thermophilus	17	0.3457	−0.6010	0.1907	0.0058
	*Neonectria_candida*	18	0.1834	−0.6190	0.2156	0.0102
	*Pichia_kluyveri*	16	0.3211	−0.4299	0.1481	0.0104
	*Oidiodendron_truncatum*	17	0.3488	0.6676	0.2444	0.0142
	*Unidentified*/k__Fungi;p__Olpidiomycota;c__Olpidiomycetes;o__Olpidiales;f__unidentified;g__unidentified;s__unidentified;	17	0.1568	−0.8076	0.3105	0.0186
	*Vishniacozyma_victoriae*	17	0.1889	−0.6696	0.2599	0.0196
	*Penicillium_pimiteouiense*	17	0.3329	−0.4693	0.1855	0.0216
	*Unidentified*/k__Fungi;p__Ascomycota;c__Sordariomycetes;o__Glomerellales;f__Plectosphaerellaceae;g__unidentified;s__unidentified;	17	0.2170	−0.7604	0.3030	0.0225
	*Debaryomyces_udenii*	17	0.2666	−0.6988	0.2822	0.0241
	*Unidentified*/k__Fungi;p__Neocallimastigomycota;c__Neocallimastigomycetes;o__Neocallimastigales;f__Neocallimastigaceae;g__unidentified;s__unidentified;	17	0.2300	0.2260	0.0970	0.0325
	Malassezia_sympodialis	17	0.0633	−0.1761	0.0777	0.0368
	*Unidentified*/k__Fungi;p__Basidiomycota;c__Agaricomycetes;o__Agaricales;f__Amanitaceae;g__Amanita;s__unidentified;	17	0.2135	0.0476	0.0213	0.0396

## Supplementary Materials

The following are available online at https://www.mdpi.com/article/10.3390/microorganisms9040741/s1, Table S1: Ratios in GID compared to CTRL: top 5 most significant ratios at each level; Table S2: Bacterial Ratios in NDD compared to GID: top 5 most significant ratios at each level; Video S1: Video Abstract: Bacterial and Fungal Dysbiosis in disorders of the Gut-Brain axis.

## Appendix A

**Table A1:** Significant changes in abundance of the mycobiome community in NDD are observed at all taxa levels.

Level	NDD Absolute Abundance Compared to CTRL	Name	Basic Covariates					Adding Probiotics	Adding Diet	Adding Probiotics and Diet
			DF	R-Squared	Beta	SEbeta	*p*-Value	*p*-Value 1	*p*-Value 2	*p*-Value 3
Phylum	Chytridiomycota	k__Fungi;p__Chytridiomycota;	14	0.4210	−1.8554	0.5119	0.0028	0.0019	0.0029	0.0013
Class	Rhizophydiomycetes	k__Fungi;p__Chytridiomycota;c__Rhizophydiomycetes;	14	0.4610	−1.7178	0.4920	0.0036	0.0249	NS	0.0111
	Orbiliomycetes	k__Fungi;p__Ascomycota;c__Orbiliomycetes;	14	0.2538	0.7096	0.3110	0.0387	NS	NS	NS
	Pezizomycetes	k__Fungi;p__Ascomycota;c__Pezizomycetes;	14	0.1742	−1.3952	0.6216	0.0415	0.0144	0.0378	0.0073
	Ustilaginomycetes	k__Fungi;p__Basidiomycota;c__Ustilaginomycetes;	14	0.1186	1.6869	0.7778	0.0478	NS	0.0538	NS
	Spizellomycetes	k__Fungi;p__Chytridiomycota;c__Spizellomycetes;	12	0.2570	−1.8647	0.7718	NS	0.0325	NS	0.0496
	Agaricomycetes	k__Fungi;p__Basidiomycota;c__Agaricomycetes;	12	0.7201	−1.1365	0.4985	NS	0.0417	NS	NS
	Eurotiomycetes	k__Fungi;p__Ascomycota;c__Eurotiomycetes;	13	0.1476	0.7961	0.3702	NS	NS	0.0509	NS
	unidentified	k__Fungi;p__Ascomycota;c__unidentified;	11	0.2480	−1.7915	0.7848	NS	NS	NS	0.0433
Family	*Alphamycetaceae*	k__Fungi;p__Chytridiomycota;c__Rhizophydiomycetes;o__Rhizophydiales;f__Alphamycetaceae;	14	0.3128	−1.1437	0.3700	0.0080		0.0042	0.0510
	*unidentified*	k__Fungi;p__Ascomycota;c__Leotiomycetes;o__unidentified;f__unidentified;	14	0.4182	1.3737	0.4703	0.0112	0.0264	0.0180	0.0499
	*unidentified*	k__Fungi;p__Chytridiomycota;c__Rhizophydiomycetes;o__Rhizophydiales;f__unidentified;	14	0.3771	−1.3958	0.5048	0.0152	0.0215	0.0164	0.0183
	*Nectriaceae*	k__Fungi;p__Ascomycota;c__Sordariomycetes;o__Hypocreales;f__Nectriaceae;	14	0.3147	1.6213	0.6036	0.0177	NS	0.0253	NS
	*Valsaceae*	k__Fungi;p__Ascomycota;c__Sordariomycetes;o__Diaporthales;f__Valsaceae;	14	0.3067	1.1744	0.4458	0.0196	NS	0.0239	NS
	*Sordariales_fam_Incertae_sedis*	k__Fungi;p__Ascomycota;c__Sordariomycetes;o__Sordariales;f__Sordariales_fam_Incertae_sedis;	14	0.3809	0.2410	0.0986	0.0284	NS	NS	NS
	*Entolomataceae*	k__Fungi;p__Basidiomycota;c__Agaricomycetes;o__Agaricales;f__Entolomataceae;	14	0.2524	−1.8388	0.7595	0.0296	0.0316	0.0306	0.0255
	*Glomerellaceae*	k__Fungi;p__Ascomycota;c__Sordariomycetes;o__Glomerellales;f__Glomerellaceae;	14	0.2860	1.5580	0.6711	0.0358	NS	0.0406	NS
	*Ustilaginaceae*	k__Fungi;p__Basidiomycota;c__Ustilaginomycetes;o__Ustilaginales;f__Ustilaginaceae;	14	0.1186	1.6869	0.7778	0.0478	NS	0.0538	NS
	*unidentified*	k__Fungi;p__Basidiomycota;c__Agaricomycetes;o__Auriculariales;f__unidentified;	12	0.3450	−2.1705	0.6456	NS	0.0057	NS	0.0124
	*unidentified*	k__Fungi;p__Ascomycota;c__Leotiomycetes;o__Helotiales;f__unidentified;	12	0.4483	−0.7760	0.2780	NS	0.0163	NS	0.0160
	*Herpotrichiellaceae*	k__Fungi;p__Ascomycota;c__Eurotiomycetes;o__Chaetothyriales;f__Herpotrichiellaceae;	12	0.2111	2.3400	0.9128	NS	0.0248	NS	0.0304
	*Halosphaeriaceae*	k__Fungi;p__Ascomycota;c__Sordariomycetes;o__Microascales;f__Halosphaeriaceae;	12	0.2201	1.7823	0.7234	NS	0.0298	NS	0.0246
	*unidentified*	k__Fungi;p__Ascomycota;c__Sordariomycetes;o__Branch06;f__unidentified;	12	0.3074	−1.8874	0.7661	NS	0.0298	NS	0.0428
	*unidentified*	k__Fungi;p__Ascomycota;c__Eurotiomycetes;o__Onygenales;f__unidentified;	12	0.2042	1.1675	0.4939	NS	0.0358	NS	0.0266
	*Tetragoniomycetaceae*	k__Fungi;p__Basidiomycota;c__Tremellomycetes;o__Trichosporonales;f__Tetragoniomycetaceae;	12	0.1551	2.3156	0.9845	NS	0.0366	NS	0.0413
	*Omphalotaceae*	k__Fungi;p__Basidiomycota;c__Agaricomycetes;o__Agaricales;f__Omphalotaceae;	12	0.1551	2.3156	0.9845	NS	0.0366	NS	0.0413
	*Dipodascaceae*	k__Fungi;p__Ascomycota;c__Saccharomycetes;o__Saccharomycetales;f__Dipodascaceae;	12	0.0943	−0.1554	0.0662	NS	0.0368	NS	0.0438
	*Cucurbitariaceae*	k__Fungi;p__Ascomycota;c__Dothideomycetes;o__Pleosporales;f__Cucurbitariaceae;	12	0.1366	1.5240	0.6699	NS	0.0421	NS	0.0469
	*Sordariales_fam_Incertae_sedis*	k__Fungi;p__Ascomycota;c__Sordariomycetes;o__Sordariales;f__Sordariales_fam_Incertae_sedis;	13	0.3350	0.2445	0.1040	NS	NS	0.0351	NS
	*Plectosphaerellaceae*	k__Fungi;p__Ascomycota;c__Sordariomycetes;o__Glomerellales;f__Plectosphaerellaceae;	11	0.3344	−1.5347	0.6468	NS	NS	NS	0.0370
	*unidentified*	k__Fungi;p__Ascomycota;c__unidentified;o__unidentified;f__unidentified;	11	0.2480	−1.7915	0.7848	NS	NS	NS	0.0433
Order	Rhizophydiales	k__Fungi;p__Chytridiomycota;c__Rhizophydiomycetes;o__Rhizophydiales;	14	0.4610	−1.7178	0.4920	0.0036	0.0249	0.0017	0.0111
	unidentified	k__Fungi;p__Ascomycota;c__Leotiomycetes;o__unidentified;	14	0.4182	1.3737	0.4703	0.0112	0.0264	0.0180	0.0499
	Diaporthales	k__Fungi;p__Ascomycota;c__Sordariomycetes;o__Diaporthales;	14	0.3451	1.4422	0.4998	0.0120	0.0534	0.0146	NS
	Orbiliales	k__Fungi;p__Ascomycota;c__Orbiliomycetes;o__Orbiliales;	14	0.3375	0.4525	0.1919	0.0334	NS	0.0520	NS
	Pezizales	k__Fungi;p__Ascomycota;c__Pezizomycetes;o__Pezizales;	14	0.1964	−1.3952	0.6162	0.0400	0.0149	0.0352	0.0070
	Ustilaginales	k__Fungi;p__Basidiomycota;c__Ustilaginomycetes;o__Ustilaginales;	14	0.1186	1.6869	0.7778	0.0478	NS	0.0538	NS
	Auriculariales	k__Fungi;p__Basidiomycota;c__Agaricomycetes;o__Auriculariales;	12	0.3452	−2.1724	0.6460	NS	0.0056	NS	0.0124
	Branch06	k__Fungi;p__Ascomycota;c__Sordariomycetes;o__Branch06;	12	0.3074	−1.8874	0.7661	NS	0.0298	NS	0.0428
	Spizellomycetales	k__Fungi;p__Chytridiomycota;c__Spizellomycetes;o__Spizellomycetales;	12	0.2570	−1.8647	0.7718	NS	0.0325	NS	0.0496
	Chaetothyriales	k__Fungi;p__Ascomycota;c__Eurotiomycetes;o__Chaetothyriales;	12	0.1421	2.1636	0.9841	NS	0.0483	NS	NS
	Leotiomycetes_ord_Incertae_sedis	k__Fungi;p__Ascomycota;c__Leotiomycetes;o__Leotiomycetes_ord_Incertae_sedis;	11	0.1224	−2.1509	0.8922	NS	NS	NS	0.0346
	unidentified	k__Fungi;p__Ascomycota;c__unidentified;o__unidentified;	11	0.2480	−1.7915	0.7848	NS	NS	NS	0.0433
*Genus*	*Fusarium*	k__Fungi;p__Ascomycota;c__Sordariomycetes;o__Hypocreales;f__Nectriaceae;g__Fusarium;	14	0.4268	1.8384	0.5123	0.0030	0.0358	0.0051	NS
	*Saccharomyces*	k__Fungi;p__Ascomycota;c__Saccharomycetes;o__Saccharomycetales;f__Saccharomycetaceae;g__Saccharomyces;	14	0.4150	1.1286	0.3333	0.0044	0.0474	0.0069	NS
	*Betamyces*	k__Fungi;p__Chytridiomycota;c__Rhizophydiomycetes;o__Rhizophydiales;f__Alphamycetaceae;g__Betamyces;	14	0.3128	−1.1437	0.3700	0.0080		0.0042	0.0510
	*unidentified*	k__Fungi;p__Ascomycota;c__Leotiomycetes;o__unidentified;f__unidentified;g__unidentified;	14	0.4182	1.3737	0.4703	0.0112	0.0264	0.0180	0.0499
	*unidentified*	k__Fungi;p__Ascomycota;c__Sordariomycetes;o__Diaporthales;f__Valsaceae;g__unidentified;	14	0.3207	1.9895	0.7075	0.0139		0.0166	NS
	*unidentified*	k__Fungi;p__Chytridiomycota;c__Rhizophydiomycetes;o__Rhizophydiales;f__unidentified;g__unidentified;	14	0.3771	−1.3958	0.5048	0.0152	0.0215	0.0164	0.0183
	*Staphylotrichum*	k__Fungi;p__Ascomycota;c__Sordariomycetes;o__Sordariales;f__Sordariales_fam_Incertae_sedis;g__Staphylotrichum;	14	0.5841	0.7721	0.2800	0.0154	0.0454	0.0210	NS
	*Engyodontium*	k__Fungi;p__Ascomycota;c__Ascomycota_cls_Incertae_sedis;o__Ascomycota_ord_Incertae_sedis;f__Ascomycota_fam_Incertae_sedis;g__Engyodontium;	14	0.3469	−0.7645	0.2794	0.0161	NS	0.0116	NS
	*Colletotrichum*	k__Fungi;p__Ascomycota;c__Sordariomycetes;o__Glomerellales;f__Glomerellaceae;g__Colletotrichum;	14	0.2860	1.5580	0.6711	0.0358	NS	0.0406	NS
	*Neonectria*	k__Fungi;p__Ascomycota;c__Sordariomycetes;o__Hypocreales;f__Nectriaceae;g__Neonectria;	14	0.1046	1.6340	0.7143	0.0382	NS	0.0533	NS
	*Melanophyllum*	k__Fungi;p__Basidiomycota;c__Agaricomycetes;o__Agaricales;f__Agaricaceae;g__Melanophyllum;	14	0.2073	1.7648	0.7733	0.0386	0.0156	0.0468	0.0190
	*unidentified*	k__Fungi;p__Mucoromycota;c__Mucoromycetes;o__Mucorales;f__Lichtheimiaceae;g__unidentified;	14	0.0957	0.0873	0.0391	0.0423	NS	0.0463	NS
	*unidentified*	k__Fungi;p__Basidiomycota;c__Agaricomycetes;o__Auriculariales;f__unidentified;g__unidentified;	11	0.3016	−2.0728	0.6945	NS	0.0057	NS	0.0124
	*unidentified*	k__Fungi;p__Ascomycota;c__Leotiomycetes;o__Helotiales;f__unidentified;g__unidentified;	11	0.4276	−0.8382	0.2949	NS	0.0163	NS	0.0160
	*Mortierella*	k__Fungi;p__Mortierellomycota;c__Mortierellomycetes;o__Mortierellales;f__Mortierellaceae;g__Mortierella;	11	0.4528	−1.7326	0.6124	NS	0.0222	NS	0.0164
	*unidentified*	k__Fungi;p__Ascomycota;c__Sordariomycetes;o__Microascales;f__Halosphaeriaceae;g__unidentified;	11	0.2080	1.9764	0.7597	NS	0.0298	NS	0.0246
	*unidentified*	k__Fungi;p__Ascomycota;c__Dothideomycetes;o__Capnodiales;f__Mycosphaerellaceae;g__unidentified;	11	0.1891	1.1674	0.4489	NS	0.0210	NS	0.0247
	*unidentified*	k__Fungi;p__Ascomycota;c__Eurotiomycetes;o__Onygenales;f__unidentified;g__unidentified;	11	0.2070	1.3142	0.5135	NS	0.0358	NS	0.0266
	*Arnium*	k__Fungi;p__Ascomycota;c__Sordariomycetes;o__Sordariales;f__Lasiosphaeriaceae;g__Arnium;	11	0.0973	2.1493	0.9062	NS	0.0277	NS	0.0370
	*Chaetosphaeronema*	k__Fungi;p__Ascomycota;c__Dothideomycetes;o__Pleosporales;f__Phaeosphaeriaceae;g__Chaetosphaeronema;	11	0.0922	2.4429	1.0354	NS	0.0273	NS	0.0379
	*Tetragoniomyces*	k__Fungi;p__Basidiomycota;c__Tremellomycetes;o__Trichosporonales;f__Tetragoniomycetaceae;g__Tetragoniomyces;	11	0.0954	2.4512	1.0611	NS	NS	NS	0.0413
	*Marasmiellus*	k__Fungi;p__Basidiomycota;c__Agaricomycetes;o__Agaricales;f__Omphalotaceae;g__Marasmiellus;	11	0.0954	2.4512	1.0611	NS	0.0366	NS	0.0413
	*Trichophaea*	k__Fungi;p__Ascomycota;c__Pezizomycetes;o__Pezizales;f__Pyronemataceae;g__Trichophaea;	11	0.0954	2.4512	1.0611	NS	0.0366	NS	0.0413
	*unidentified*	k__Fungi;p__Ascomycota;c__Sordariomycetes;o__Branch06;f__unidentified;g__unidentified;	11	0.2450	−1.9076	0.8332	NS	0.0298	NS	0.0428
	*unidentified*	k__Fungi;p__Ascomycota;c__unidentified;o__unidentified;f__unidentified;g__unidentified;	11	0.2480	−1.7915	0.7848	NS	NS	NS	0.0433
	*Exophiala*	k__Fungi;p__Ascomycota;c__Eurotiomycetes;o__Chaetothyriales;f__Herpotrichiellaceae;g__Exophiala;	11	0.0584	2.3498	1.0471	NS	0.0383	NS	0.0464
	*Pyrenochaeta*	k__Fungi;p__Ascomycota;c__Dothideomycetes;o__Pleosporales;f__Cucurbitariaceae;g__Pyrenochaeta;	11	0.0663	1.6738	0.7547	NS	0.0435	NS	0.0486
	*Plectosphaerella*	k__Fungi;p__Ascomycota;c__Sordariomycetes;o__Glomerellales;f__Plectosphaerellaceae;g__Plectosphaerella;	11	0.1903	−1.3601	0.6277	NS	NS	NS	0.0531
*Species*	*Saccharomyces_cerevisiae*	k__Fungi;p__Ascomycota;c__Saccharomycetes;o__Saccharomycetales;f__Saccharomycetaceae;g__Saccharomyces;s__Saccharomyces_cerevisiae;	14	0.4150	1.1286	0.3333	0.0044	0.0474	0.0069	NS
	*Betamyces_americae-meridionalis*	k__Fungi;p__Chytridiomycota;c__Rhizophydiomycetes;o__Rhizophydiales;f__Alphamycetaceae;g__Betamyces;s__Betamyces_americae-meridionalis;	14	0.3128	−1.1437	0.3700	0.0080	NS	0.0042	0.0510
	*unidentified*	k__Fungi;p__Ascomycota;c__Leotiomycetes;o__unidentified;f__unidentified;g__unidentified;s__unidentified;	14	0.4182	1.3737	0.4703	0.0112	0.0264	0.0180	0.0499
	*Peziza_nivalis*	k__Fungi;p__Ascomycota;c__Pezizomycetes;o__Pezizales;f__Pezizaceae;g__Peziza;s__Peziza_nivalis;	14	0.2416	0.4322	0.1520	0.0130	0.0267	0.0145	0.0260
	*unidentified*	k__Fungi;p__Ascomycota;c__Sordariomycetes;o__Diaporthales;f__Valsaceae;g__unidentified;s__unidentified;	14	0.3207	1.9895	0.7075	0.0139	NS	0.0166	NS
	*Fusarium_oxysporum*	k__Fungi;p__Ascomycota;c__Sordariomycetes;o__Hypocreales;f__Nectriaceae;g__Fusarium;s__Fusarium_oxysporum;	14	0.3128	1.4464	0.5171	0.0143	NS	0.0163	NS
	*unidentified*	k__Fungi;p__Chytridiomycota;c__Rhizophydiomycetes;o__Rhizophydiales;f__unidentified;g__unidentified;s__unidentified;	14	0.3771	−1.3958	0.5048	0.0152	0.0215	0.0164	0.0183
	*Staphylotrichum_coccosporum*	k__Fungi;p__Ascomycota;c__Sordariomycetes;o__Sordariales;f__Sordariales_fam_Incertae_sedis;g__Staphylotrichum;s__Staphylotrichum_coccosporum;	14	0.5851	0.8130	0.2963	0.0158	0.0449	0.0214	NS
	*Engyodontium_album*	k__Fungi;p__Ascomycota;c__Ascomycota_cls_Incertae_sedis;o__Ascomycota_ord_Incertae_sedis;f__Ascomycota_fam_Incertae_sedis;g__Engyodontium;s__Engyodontium_album;	14	0.3469	−0.7645	0.2794	0.0161	NS	0.0116	NS
	*Chaetomium_erectum*	k__Fungi;p__Ascomycota;c__Sordariomycetes;o__Sordariales;f__Chaetomiaceae;g__Chaetomium;s__Chaetomium_erectum;	14	0.2427	1.8071	0.7522	0.0307	0.0143	0.0378	0.0176
	*Neonectria_candida*	k__Fungi;p__Ascomycota;c__Sordariomycetes;o__Hypocreales;f__Nectriaceae;g__Neonectria;s__Neonectria_candida;	14	0.1046	1.6340	0.7143	0.0382	NS	0.0533	NS
	*Melanophyllum_haematospermum*	k__Fungi;p__Basidiomycota;c__Agaricomycetes;o__Agaricales;f__Agaricaceae;g__Melanophyllum;s__Melanophyllum_haematospermum;	14	0.2073	1.7648	0.7733	0.0386	0.0156	0.0468	0.0190
	*unidentified*	k__Fungi;p__Mucoromycota;c__Mucoromycetes;o__Mucorales;f__Lichtheimiaceae;g__unidentified;s__unidentified;	14	0.0957	0.0873	0.0391	0.0423	NS	0.0463	NS
	*Spizellomyces_dolichospermus*	k__Fungi;p__Chytridiomycota;c__Spizellomycetes;o__Spizellomycetales;f__Spizellomycetaceae;g__Spizellomyces;s__Spizellomyces_dolichospermus;	14	0.2033	−1.7153	0.7748	0.0439	0.0455	0.0501	0.0442
	*Hypochnicium_bombycinum*	k__Fungi;p__Basidiomycota;c__Agaricomycetes;o__Corticiales;f__Corticiaceae;g__Hypochnicium;s__Hypochnicium_bombycinum;	14	0.1286	0.7133	0.3367	0.0525	NS	NS	NS
	*Microascus_paisii*	k__Fungi;p__Ascomycota;c__Sordariomycetes;o__Microascales;f__Microascaceae;g__Microascus;s__Microascus_paisii;	14	0.1093	0.7894	0.3730	0.0527	NS	0.0442	NS
	*unidentified*	k__Fungi;p__Ascomycota;c__Leotiomycetes;o__Helotiales;f__unidentified;g__unidentified;s__unidentified;	12	0.4483	−0.7760	0.2780	NS	0.0163	NS	0.0160
	*unidentified*	k__Fungi;p__Basidiomycota;c__Agaricomycetes;o__Auriculariales;f__unidentified;g__unidentified;s__unidentified;	12	0.3450	−2.1705	0.6456	NS	0.0057	NS	0.0124
	*unidentified*	k__Fungi;p__Ascomycota;c__Dothideomycetes;o__Capnodiales;f__Mycosphaerellaceae;g__unidentified;s__unidentified;	12	0.2411	1.1072	0.4170	NS	0.0210	NS	0.0247
	*unidentified*	k__Fungi;p__Ascomycota;c__Dothideomycetes;o__Pleosporales;f__Phaeosphaeriaceae;g__Chaetosphaeronema;s__unidentified;	12	0.1657	2.3941	0.9530	NS	0.0273	NS	NS
	*Arnium_arizonense*	k__Fungi;p__Ascomycota;c__Sordariomycetes;o__Sordariales;f__Lasiosphaeriaceae;g__Arnium;s__Arnium_arizonense;	12	0.1686	2.0909	0.8350	NS	0.0277	NS	0.0370
	*Chaetomium_grande*	k__Fungi;p__Ascomycota;c__Sordariomycetes;o__Sordariales;f__Chaetomiaceae;g__Chaetomium;s__Chaetomium_grande;	12	0.5311	−1.3565	0.5441	NS	0.0283	NS	NS
	*unidentified*	k__Fungi;p__Ascomycota;c__Sordariomycetes;o__Microascales;f__Halosphaeriaceae;g__unidentified;s__unidentified;	12	0.2197	1.7837	0.7239	NS	0.0298	NS	0.0246
	*unidentified*	k__Fungi;p__Ascomycota;c__Sordariomycetes;o__Branch06;f__unidentified;g__unidentified;s__unidentified;	12	0.3074	−1.8874	0.7661	NS	0.0298	NS	NS
	*Phialophora_mustea*	k__Fungi;p__Ascomycota;c__Eurotiomycetes;o__Chaetothyriales;f__Herpotrichiellaceae;g__Phialophora;s__Phialophora_mustea;	12	0.1589	2.3167	0.9793	NS	0.0357	NS	0.0404
	*unidentified*	k__Fungi;p__Ascomycota;c__Eurotiomycetes;o__Onygenales;f__unidentified;g__unidentified;s__unidentified;	12	0.2042	1.1675	0.4939	NS	0.0358	NS	0.0266
	*Exophiala_dermatitidis*	k__Fungi;p__Ascomycota;c__Eurotiomycetes;o__Chaetothyriales;f__Herpotrichiellaceae;g__Exophiala;s__Exophiala_dermatitidis;	12	0.1551	2.2601	0.9609	NS	0.0366	NS	0.0413
	*Dioszegia_fristingensis*	k__Fungi;p__Basidiomycota;c__Tremellomycetes;o__Tremellales;f__Bulleribasidiaceae;g__Dioszegia;s__Dioszegia_fristingensis;	12	0.1551	2.3156	0.9845	NS	0.0366	NS	0.0413
	*Pyrenochaeta_keratinophila*	k__Fungi;p__Ascomycota;c__Dothideomycetes;o__Pleosporales;f__Cucurbitariaceae;g__Pyrenochaeta;s__Pyrenochaeta_keratinophila;	12	0.1551	1.7072	0.7258	NS	0.0366	NS	0.0413
	*Exophiala_oligosperma*	k__Fungi;p__Ascomycota;c__Eurotiomycetes;o__Chaetothyriales;f__Herpotrichiellaceae;g__Exophiala;s__Exophiala_oligosperma;	12	0.1551	2.3156	0.9845	NS	0.0366	NS	0.0413
	*Alternaria_hungarica*	k__Fungi;p__Ascomycota;c__Dothideomycetes;o__Pleosporales;f__Pleosporaceae;g__Alternaria;s__Alternaria_hungarica;	12	0.1551	0.5971	0.2539	NS	0.0366	NS	0.0413
	*Peziza_buxea*	k__Fungi;p__Ascomycota;c__Pezizomycetes;o__Pezizales;f__Pezizaceae;g__Peziza;s__Peziza_buxea;	12	0.1551	2.3156	0.9845	NS	0.0366	NS	0.0413
	*Tetragoniomyces_uliginosus*	k__Fungi;p__Basidiomycota;c__Tremellomycetes;o__Trichosporonales;f__Tetragoniomycetaceae;g__Tetragoniomyces;s__Tetragoniomyces_uliginosus;	12	0.1551	2.3156	0.9845	NS	0.0366	NS	0.0413
	*unidentified*	k__Fungi;p__Ascomycota;c__Dothideomycetes;o__Pleosporales;f__Leptosphaeriaceae;g__Leptosphaeria;s__unidentified;	12	0.1551	2.3152	0.9843	NS	0.0366	NS	0.0413
	*unidentified*	k__Fungi;p__Basidiomycota;c__Agaricomycetes;o__Agaricales;f__Omphalotaceae;g__Marasmiellus;s__unidentified;	12	0.1551	2.3156	0.9845	NS	0.0366	NS	0.0413
	*Exophiala_phaeomuriformis*	k__Fungi;p__Ascomycota;c__Eurotiomycetes;o__Chaetothyriales;f__Herpotrichiellaceae;g__Exophiala;s__Exophiala_phaeomuriformis;	12	0.1551	2.3156	0.9845	NS	0.0366	NS	0.0413
	*unidentified*	k__Fungi;p__Ascomycota;c__Pezizomycetes;o__Pezizales;f__Pyronemataceae;g__Trichophaea;s__unidentified;	12	0.1551	2.3156	0.9845	NS	0.0366	NS	0.0413
	*Aspergillus_ochraceus*	k__Fungi;p__Ascomycota;c__Eurotiomycetes;o__Eurotiales;f__Aspergillaceae;g__Aspergillus;s__Aspergillus_ochraceus;	12	0.1551	1.4435	0.6137	NS	0.0366	NS	0.0413
	*Mortierella_zonata*	k__Fungi;p__Mortierellomycota;c__Mortierellomycetes;o__Mortierellales;f__Mortierellaceae;g__Mortierella;s__Mortierella_zonata;	12	0.1551	2.3156	0.9845	NS	0.0366	NS	0.0413
	*unidentified*	k__Fungi;p__Ascomycota;c__Eurotiomycetes;o__Chaetothyriales;f__Herpotrichiellaceae;g__Phialophora;s__unidentified;	12	0.1551	2.3156	0.9845	NS	0.0366	NS	0.0413
	*Microascus_albonigrescens*	k__Fungi;p__Ascomycota;c__Sordariomycetes;o__Microascales;f__Microascaceae;g__Microascus;s__Microascus_albonigrescens;	12	0.1551	2.3156	0.9845	NS	0.0366	NS	0.0413
	*Coniophora_olivacea*	k__Fungi;p__Basidiomycota;c__Agaricomycetes;o__Boletales;f__Coniophoraceae;g__Coniophora;s__Coniophora_olivacea;	12	0.1522	2.3083	0.9868	NS	0.0374	NS	0.0420
	*Mortierella_amoeboidea*	k__Fungi;p__Mortierellomycota;c__Mortierellomycetes;o__Mortierellales;f__Mortierellaceae;g__Mortierella;s__Mortierella_amoeboidea;	12	0.3633	−1.2981	0.5824	NS	0.0457	NS	0.0549
	*unidentified*	k__Fungi;p__Mortierellomycota;c__Mortierellomycetes;o__Mortierellales;f__Mortierellaceae;g__Mortierella;s__unidentified;	12	0.3202	−1.1002	0.5135	NS	0.0534	NS	0.0140
	*unidentified*	k__Fungi;p__Basidiomycota;c__Wallemiomycetes;o__Wallemiales;f__Wallemiales_fam_Incertae_sedis;g__Wallemia;s__unidentified;	12	0.6577	−1.1381	0.5338	NS	0.0544	NS	NS
	*unidentified*	k__Fungi;p__Ascomycota;c__Sordariomycetes;o__Branch06;f__unidentified;g__unidentified;s__unidentified;	11	0.2450	−1.9076	0.8332	NS	NS	NS	0.0428
	*unidentified*	k__Fungi;p__Ascomycota;c__unidentified;o__unidentified;f__unidentified;g__unidentified;s__unidentified;	11	0.2480	−1.7915	0.7848	NS	NS	NS	0.0433
	*Plectosphaerella_cucumerina*	k__Fungi;p__Ascomycota;c__Sordariomycetes;o__Glomerellales;f__Plectosphaerellaceae;g__Plectosphaerella;s__Plectosphaerella_cucumerina;	11	0.1903	−1.3601	0.6277	NS	NS	NS	0.0531
	*unidentified*	k__Fungi;p__Ascomycota;c__Dothideomycetes;o__Pleosporales;f__Phaeosphaeriaceae;g__Chaetosphaeronema;s__unidentified;	11	0.0922	2.4429	1.0354	NS	NS	NS	0.0379
