# Supplementary material for: A Comparative Pilot Study of Bacterial and Fungal Dysbiosis in Neurodevelopmental Disorders and Gastrointestinal Disorders: Commonalities, Specificities and Correlations with Lifestyle
Source: Microorganisms. 2021 Apr 2;9(4):741. doi: 10.3390/microorganisms9040741 (PMC8065742; doi:10.3390/microorganisms9040741)
Supplement: Supplementary file 1 [file microorganisms-09-00741-s001.zip › supplementary tables.docx]

SUPPLEMENTARY TABLES

Table S1. Ratios in GID compared to CTRL: top 5 most significant ratios at each level

| Level |  |  | basic covariates + probiotics and diet | | | | |
| --- | --- | --- | --- | --- | --- | --- | --- |
|  |  |  | DF | R-squared | beta | SEbeta | P.value |
| Phylum | ↓ | TM7/Actinobacteria | 42 | 0.1249 | -0.0076 | 0.0031 | 0.0178 |
|  | ↑ | Firmicutes/Verrucomicrobia | 38 | 0.1087 | 6035.7042 | 2508.9043 | 0.0211 |
|  | ↑ | Proteobacteria/Verrucomicrobia | 38 | 0.1934 | 756.3134 | 320.3748 | 0.0235 |
|  | ↓ | Firmicutes/Actinobacteria | 42 | 0.2291 | -106.9746 | 49.1642 | 0.0352 |
|  | ↑ | Cyanobacteria/Verrucomicrobia | 38 | 0.0704 | 4.2385 | 1.9951 | 0.0402 |
| Class | ↑ | Erysipelotrichi/Verrucomicrobiae | 28 | 0.2149 | 149.6008 | 49.0335 | 0.0049 |
|  | ↑ | Clostridia/Verrucomicrobiae | 28 | 0.1804 | 9391.7048 | 3234.7252 | 0.0071 |
|  | ↑ | Coriobacteriia/Verrucomicrobiae | 28 | 0.1582 | 163.5255 | 61.2332 | 0.0125 |
|  | ↑ | Chloroplast/Verrucomicrobiae | 28 | 0.1530 | 6.8428 | 2.6269 | 0.0145 |
|  | ↑ | Betaproteobacteria/Chloroplast | 15 | 0.1553 | 301.9490 | 119.5450 | 0.0233 |
| Family | ↑ | Alcaligenaceae/[Odoribacteraceae] | 30 | 0.4120 | 26.0400 | 6.4763 | 0.0004 |
|  | ↑ | [Mogibacteriaceae]/Verrucomicrobiaceae | 28 | 0.2130 | 3.2079 | 0.9997 | 0.0033 |
|  | ↑ | Erysipelotrichaceae/Verrucomicrobiaceae | 28 | 0.2149 | 149.6008 | 49.0335 | 0.0049 |
|  | ↑ | Streptococcaceae/Verrucomicrobiaceae | 28 | 0.1666 | 68.3941 | 23.4209 | 0.0068 |
|  | ↑ | Enterococcaceae/Verrucomicrobiaceae | 28 | 0.1607 | 1.8147 | 0.6303 | 0.0076 |
| Order | ↑ | Erysipelotrichales/Verrucomicrobiales | 28 | 0.2149 | 149.6008 | 49.0335 | 0.0049 |
|  | ↑ | Clostridiales/Verrucomicrobiales | 28 | 0.1804 | 9391.7048 | 3234.7252 | 0.0071 |
|  | ↑ | Coriobacteriales/Verrucomicrobiales | 28 | 0.1582 | 163.5255 | 61.2332 | 0.0125 |
|  | ↑ | Streptophyta/Verrucomicrobiales | 28 | 0.1530 | 6.8428 | 2.6269 | 0.0145 |
|  | ↑ | Enterobacteriales/Verrucomicrobiales | 28 | 0.1798 | 375.5898 | 144.3047 | 0.0146 |
| Genus | ↑ | Anaerotruncus/Others | 31 | 0.2575 | 0.0010 | 0.0003 | 0.0015 |
|  | ↑ | Anaerotruncus/Roseburia | 31 | 0.2013 | 0.0118 | 0.0034 | 0.0016 |
|  | ↑ | Butyricicoccus/Akkermansia | 28 | 0.1533 | 60.6300 | 18.3339 | 0.0026 |
|  | ↑ | Eggerthella/Blautia | 31 | 0.2400 | 0.0354 | 0.0114 | 0.0040 |
|  | ↑ | Anaerotruncus/Akkermansia | 28 | 0.2556 | 2.8920 | 0.9268 | 0.0042 |
| Species | ↑ | pullicaecorum/muciniphila | 28 | 0.1737 | 63.6225 | 18.5312 | 0.0019 |
|  | ↑ | mucosae/eutactus | 14 | 0.2804 | 0.0094 | 0.0028 | 0.0046 |
|  | ↑ | lenta/formicigenerans | 29 | 0.1684 | 1.1798 | 0.4036 | 0.0067 |
|  | ↑ | aureum/formicigenerans | 29 | 0.1780 | 0.0128 | 0.0045 | 0.0082 |
|  | ↑ | distasonis/catus | 29 | 0.3013 | 36.5526 | 13.6593 | 0.0121 |

^1^Arrows show the increase or decrease of the specified ratios in GID group compared to CTRL.

Table S2. Bacterial Ratios in NDD compared to GID: top 5 most significant ratios at each level

| Level | Ratio in NDD compared to GID (top 10 most significant ineach level) | basic covariates + probiotics and diet | | | | |
| --- | --- | --- | --- | --- | --- | --- |
|  |  | DF | R-squared | beta | SEbeta | P.value |
| Phylum | TM7/Actinobacteria | 42 | 0.1249 | -0.0076 | 0.0031 | 0.0178 |
|  | Firmicutes/Verrucomicrobia | 38 | 0.1087 | 6035.7042 | 2508.9043 | 0.0211 |
|  | Proteobacteria/Verrucomicrobia | 38 | 0.1934 | 756.3134 | 320.3748 | 0.0235 |
|  | Firmicutes/Actinobacteria | 42 | 0.2291 | -106.9746 | 49.1642 | 0.0352 |
| Class | Coriobacteriia/Verrucomicrobiae | 38 | 0.1332 | 131.1109 | 48.1861 | 0.0098 |
|  | Gammaproteobacteria/Verrucomicrobiae | 38 | 0.1395 | 551.8550 | 205.1041 | 0.0105 |
|  | Bacteroidia/Chloroplast | 24 | 0.3169 | 7705.5353 | 2816.2208 | 0.0115 |
|  | Clostridia/Verrucomicrobiae | 38 | 0.1093 | 5847.6557 | 2459.9694 | 0.0226 |
|  | Erysipelotrichi/Verrucomicrobiae | 38 | 0.0534 | 141.1575 | 59.3833 | 0.0226 |
| Family | Alcaligenaceae/[Odoribacteraceae] | 40 | 0.2696 | 23.5112 | 6.6239 | 0.0010 |
|  | Enterobacteriaceae/Verrucomicrobiaceae | 38 | 0.1691 | 573.2619 | 197.1120 | 0.0060 |
|  | Coriobacteriaceae/Verrucomicrobiaceae | 38 | 0.1332 | 131.1109 | 48.1861 | 0.0098 |
|  | Lachnospiraceae/Verrucomicrobiaceae | 38 | 0.1357 | 2923.5207 | 1212.4784 | 0.0208 |
|  | Erysipelotrichaceae/Verrucomicrobiaceae | 38 | 0.0534 | 141.1575 | 59.3833 | 0.0226 |
| Order | Enterobacteriales/Verrucomicrobiales | 38 | 0.1691 | 573.2619 | 197.1120 | 0.0060 |
|  | Coriobacteriales/Verrucomicrobiales | 38 | 0.1332 | 131.1109 | 48.1861 | 0.0098 |
|  | Gemellales/Streptophyta | 24 | 0.1519 | 0.2820 | 0.1029 | 0.0114 |
|  | Bacteroidales/Streptophyta | 24 | 0.3169 | 7705.5353 | 2816.2208 | 0.0115 |
|  | Clostridiales/Verrucomicrobiales | 38 | 0.1093 | 5847.6557 | 2459.9694 | 0.0226 |
| Genus | Butyricicoccus/Akkermansia | 38 | 0.1440 | 53.6260 | 16.8430 | 0.0029 |
|  | Ruminococcus/Anaerotruncus | 29 | 0.2546 | -645.2064 | 205.3775 | 0.0039 |
|  | Odoribacter/Anaerotruncus | 29 | 0.1815 | -53.2325 | 17.4606 | 0.0049 |
|  | Escherichia/Akkermansia | 38 | 0.1695 | 331.8165 | 116.4929 | 0.0071 |
|  | Eggerthella/Blautia | 42 | 0.1272 | 0.0363 | 0.0132 | 0.0086 |
| Species | pullicaecorum/muciniphila | 38 | 0.1520 | 55.8348 | 16.9841 | 0.0022 |
|  | coli/muciniphila | 38 | 0.1696 | 331.8584 | 116.4884 | 0.0070 |
|  | lenta/formicigenerans | 38 | 0.1339 | 0.8926 | 0.3181 | 0.0079 |
|  | aureum/longum | 36 | 0.1518 | 0.0020 | 0.0008 | 0.0205 |
|  | catus/longum | 36 | 0.1407 | -8.0524 | 3.3314 | 0.0208 |

^1^Arrows show the increase or decrease of the specified ratios in NDD group compared to GID.
